# Impact of Nanoparticle Stiffness on Endosomal Escape and Signaling Pathways in Cytosolic Delivery

**DOI:** 10.1002/adhm.202501706

**Published:** 2025-07-14

**Authors:** Yali Zhang, Yue Hui, Zichao Guo, Dawei Liu, Yun Liu, Huajian Gao, Chun‐Xia Zhao

**Affiliations:** ^1^ School of Chemical Engineering University of Adelaide Adelaide 5005 Australia; ^2^ Mechano‐X Institute Applied Mechanics Laboratory Department of Engineering Mechanics Tsinghua University Beijing 100084 China

**Keywords:** drug delivery, endosomal escape, nanoparticles, signaling pathway, stiffness

## Abstract

Viruses utilize stiffness tuning to enhance cell entry and uncoating, as cells can regulate the uptake process by sensing the mechanical stimulation of particles. The improved cytosolic delivery efficiency enhances the nanoparticle (NPs) accumulation in target sites, which is a prerequisite for achieving efficient treatment performance. However, the preparation of NPs with similar physicochemical properties but distinct stiffnesses is relatively limited, and the role of NP stiffness in intracellular distribution remains elusive. In this study, using two silica precursors at different molar ratios, silica nanocapsules (SNCs) are synthesized with a stiffness range (1.37 MPa to 1.72 GPa) spanning orders of magnitude. Additionally, an endosomal escape assay (EEA) is developed to enable rapid quantification of NP intracellular distribution based on cell fractionation. SNCs with lower stiffness exhibit superior cellular uptake efficiency. The hard SNCs however, demonstrate ≈1.8 fold‐enhanced endosomal escape efficiency compared to soft SNCs. Sequencing results reveal that SNCs with higher stiffness activate the reactive oxygen species (ROS)‐mediated mechanism, which facilitates rapid endosomal escape by inducing moderate oxidative stress. This work highlights the critical role of NP stiffness in regulating cytosolic delivery and the trade‐off between nanotoxicity and delivery efficiency.

## Graphical abstract

Nanoparticle (NP) stiffness affects cellular uptake, but its impact on intracellular distribution remains unclear. This study synthesizes silica nanocapsules with varying stiffness, inspired by viral mechanisms, and applies assays to measure cellular uptake and escape efficiency. Findings highlight the crucial role of NP stiffness in regulating cytosolic delivery and the trade‐off between nanotoxicity and delivery efficiency.

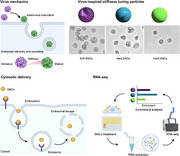

## 1 Introduction

Nanoparticle (NP) based drug delivery systems have advanced cancer therapy compared with conventional formulations by controlling drug release, delivering therapeutics to target sites in a controlled manner, and facilitating drug combinations with imaging capabilities.^[^ ^1^, ^2^, ^3^ ^]^ Additionally, NPs exhibit lower immunogenicity, customizable material composition, and easier, more cost‐effective production for clinical transition.^[^ ^4^, ^5^, ^6^ ^]^ Despite these characteristics, inadequate cytosolic delivery of therapeutics remains a crucial challenge for achieving efficient delivery at target sites.^[^ ^2^ ^]^

Viruses have shown improved cell entry and cytosolic delivery, for example, by tuning their mechanical properties to infect and invade host cells.^[^ ^7^, ^8^, ^9^ ^]^ A cytoplasmic tail domain‐mediated stiffness switch has been observed in human immunodeficiency viruses (HIV) during maturation.^[^ ^8^ ^]^ Adenoviruses (AdV), however, decondense their genetic material during maturation to increase internal pressure and virion stiffness.^[^ ^9^, ^10^ ^]^ This increased stiffness likely reduces the resilience of virions, promoting the biophysical destabilization of pentons and facilitating the disassembly of mature AdV.

Emerging studies have shown that NP stiffness exhibits a great influence on endocytosis efficiency.^[^ ^11^, ^12^, ^13^, ^14^, ^15^, ^16^, ^17^, ^18^ ^]^ For instance, in HeLa cancer cells, stiff lipid NPs (1.2 GPa) exhibited superior uptake compared to softer counterparts (0.76 GPa).^[^ ^19^ ^]^ Teng et al. explored the uptake efficiency of PEG‐conjugated silica NPs with low and high stiffness (Young's modulus of 233 and 47 MPa) in MCF‐7 breast cancer cells. Their results reveal that soft NPs demonstrated up to 26 fold higher uptake efficiency than hard NPs.^[^ ^20^ ^]^ While conflicting findings may arise from the affinity of different nanomaterials (e.g., liposomes, hydrogels and silica NPs), conjugated ligands and various cell models, NP stiffness, in essence is a key parameter regulating internalization effectiveness.

So far, the role of NP stiffness in mediating intracellular delivery remains unclear. Specifically, after endocytosis, how does NP stiffness affect endosomal escape and cytosolic delivery? Are there any cell responses specific to NP stiffness? Addressing these questions could provide insights for improving NP design and delivery strategies. However, the challenge of synthesizing NPs with distinct stiffness while maintaining similar physicochemical properties (e.g., size, shape, and charge) limits the exploration of NPs stiffness in drug delivery. The stiffness of hydrogel NPs, for example, can be adjusted by manipulating the crosslinker ratios, providing Young's moduli ranging only from kPa to MPa.^[^ ^19^ ^]^ Silica has shown excellent tuning of mechanical properties due to its internal Si‐O‐Si structure, which provides the possibility to encompass a broad range of stiffness.^[^ ^18^, ^21^ ^]^

**Figure 1:**
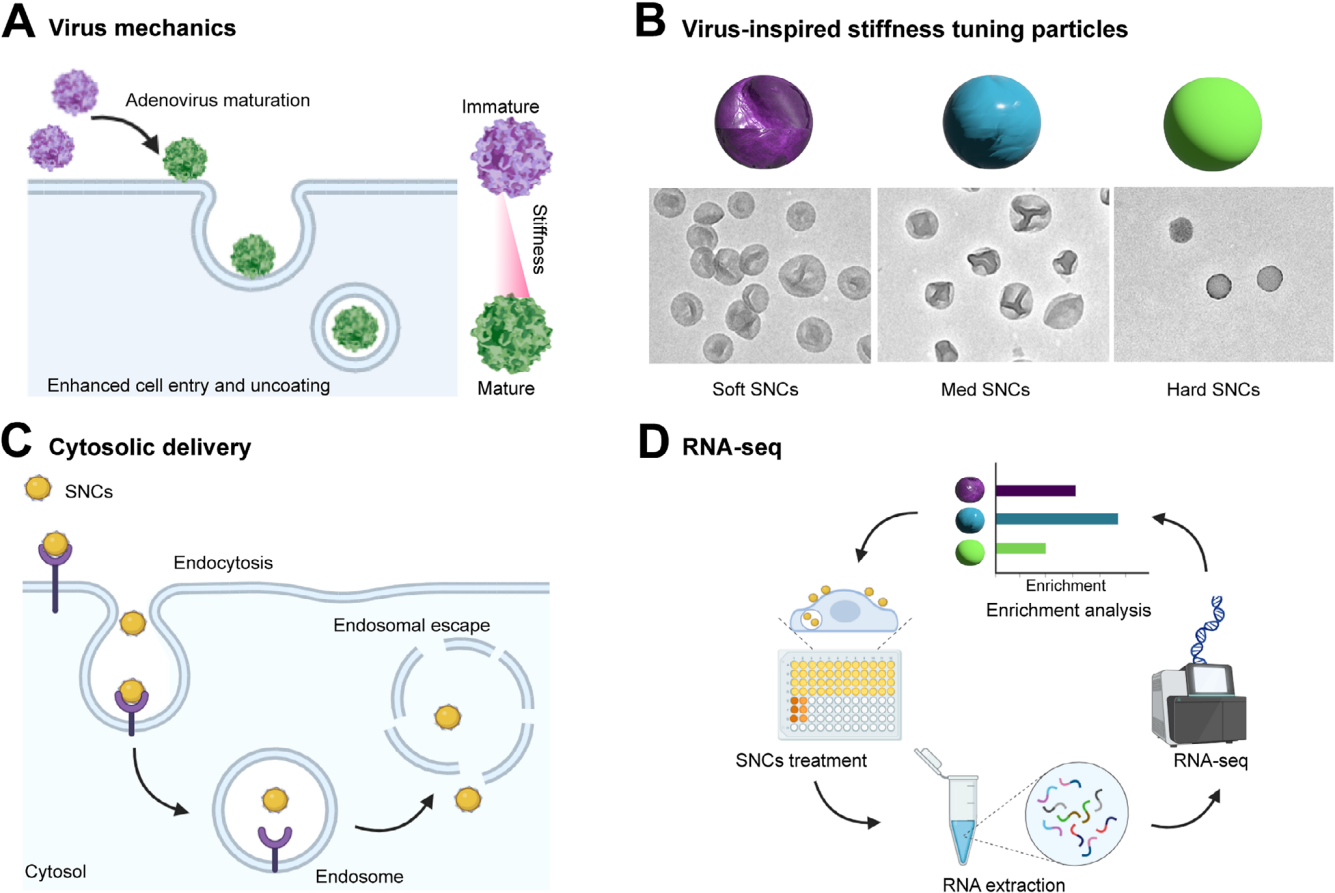
Overview of the study design and workflow. Procedures used to synthesize viral mechanism‐inspired A) SNCs with tunable stiffness B), develop assays to examine particle uptake and cytosolic delivery efficiency C), and identify potential molecular mechanisms underlying stiffness‐associated cell responses D). Part of the figure was created with BioRender.com.

We have developed a protocol to synthesize silica nanocapsules (SNCs) with similar physicochemical properties but a wide range of stiffness, with Young's moduli spanning from kPa to GPa.^[^ ^18^, ^21^ ^]^ This strategy allows us to investigate the effect of NP stiffness on intracellular distribution and biological responses. Inspired by the viral stiffness‐tuning machinery (**Figure** **1**A), we hypothesized that NP stiffness influences uptake and endosomal escape capabilities through distinct cellular features and pathways.

To address these questions, we first synthesized three silica nanocapsules (SNCs) with distinct stiffness: soft, medium, and hard NPs with Young's moduli of 1.37 MPa, 13.22 MPa, and 1.72 GPa, respectively (Figure 1B). We also developed an endosomal escape assay (EEA) to quantitatively assess and compare the intracellular distribution profile, cellular uptake levels, and endosomal escape efficiency of the SNCs with distinct stiffness (Figure 1C). The hard SNCs demonstrated a 1.8 fold increase in endosomal escape efficiency (% uptake) over soft SNCs within 4 h, despite the latter showing greater uptake capability. Further RNA sequencing revealed distinct cell signaling pathways associated with NP stiffness (Figure 1D).

## 2 Results

### 2.1 Synthesis and Characterization of SNCs with Tunable Stiffness

**Figure 2:**
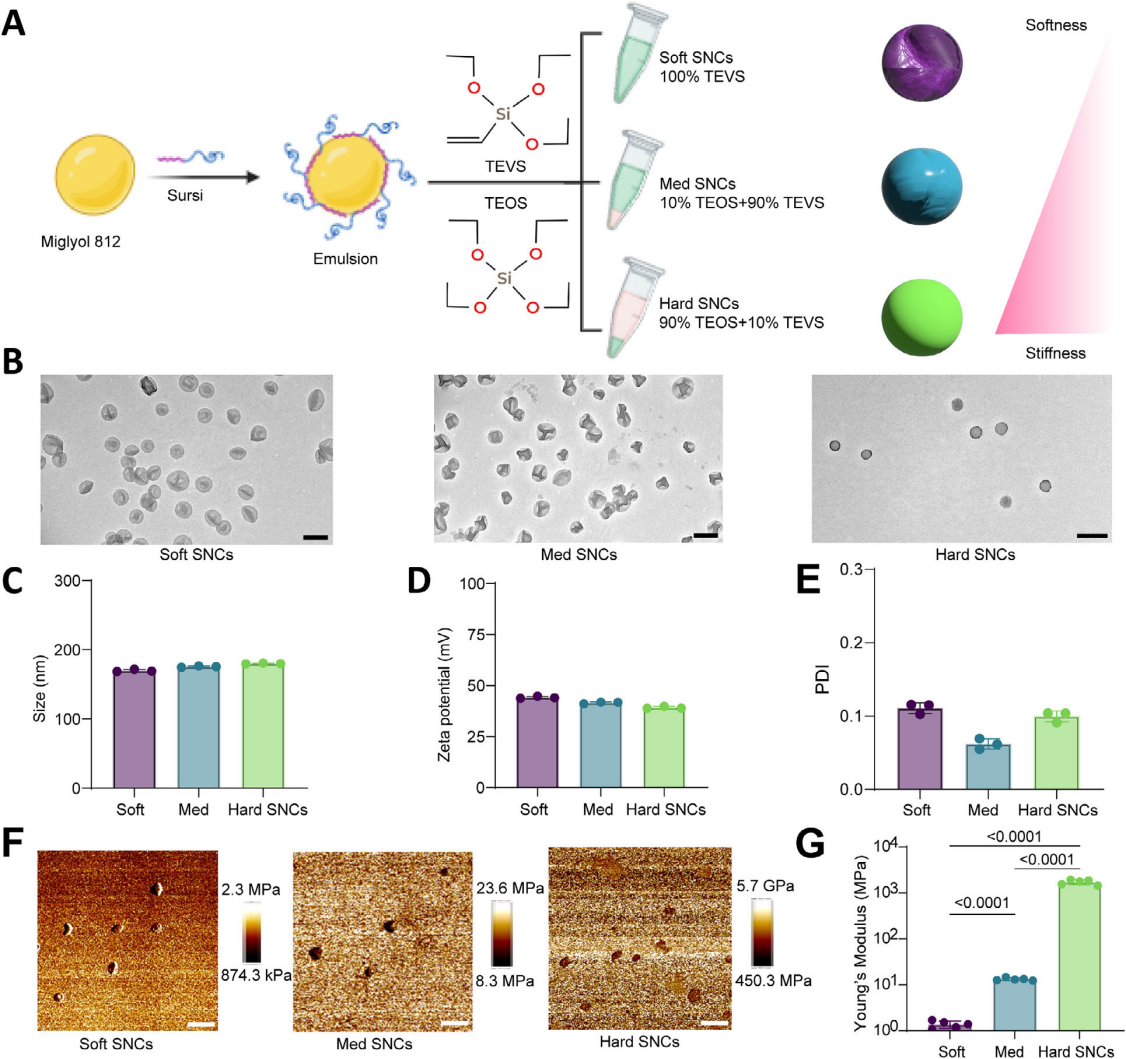
Synthesis and characterization of soft, med, and hard SNCs. A) Schematic illustration of the synthesis process of SNCs with tunable Young's moduli. B) Representative TEM images of the SNCs (scale bars, 200 nm). C) Particle size of SNCs (n = 3; mean ± SD). D) Zeta potential of SNCs (n = 3; mean ± SD). E) Polydispersity index (PDI) of SNCs (n = 3; mean ± SD). SNCs were suspended in 2.5 mm HEPES buffer for DLS analysis. F) Representative AFM profile of the SNCs (scale bars, 600 nm). G) Young's modulus of synthesized SNCs (n = 5; mean ± SD; *p* < 0.0001). n = biological replicates, each performed in technical triplicate. Statistical significance was considered at *p* < 0.05. P‐values are determined using by one‐way ANOVA followed by Tukey's multiple comparison test. Part of the figure was created with BioRender.com.

We first synthesized SNCs with tunable stiffness using a bifunctional peptide, SurSi (Ac‐MKQLAHSVSRLEHA RKKRKKRKKRKKGGGY‐CONH_2_), based on previously developed protocols (**Figure** **2**A).^[^ ^18^ ^]^ The stiffness of the particles was controlled by using two different silica precursors, triethoxyvinylsilane (TEVS) and tetraethoxysilane (TEOS), at different TEVS/TEOS molar ratios of 100, 90, and 10 mol%. These three formulations are referred to as soft (soft, 100% TEVS/0% TEOS), medium (med, 90% TEVS/10% TEOS), and hard SNCs (hard, 10% TEVS/90% TEOS) (Figure 2A).

We characterized these three SNCs using transmission electron micrography (TEM), dynamic light scattering (DLS), and atomic force microscopy (AFM). TEM images (Figure 2B) revealed the formation of spherical structures in all three SNCs. The soft and med SNCs exhibited buckled morphologies with central collapse, whereas hard SNCs maintained a uniform spherical shape. All three SNCs have a similar hydrodynamic size of ≈175 ± 10 nm with polydispersity index (PDI) of less than 0.15, and zeta (ζ) potentials of ≈40 ± 5 mV (Figure 2C–E; Table S2, Supporting Information). The highly positive zeta potential of SNCs is due to the incorporation of SurSi peptide in the silica shell, which has a polycationic tail. AFM characterization revealed that soft SNCs exhibited a Young's moduli of 1.37 ± 0.22 MPa, while med and hard SNCs had Young's moduli of 13.22 ± 0.85 MPa and 1.72 ± 0.19 GPa, respectively (Figure 2F,G). Our data demonstrate that the SNC formulations, with a board range of particle stiffness independent of other physical properties (size and charge), provide an ideal model system for examining the impact of stiffness on cellular uptake and cytosolic delivery.^[^ ^18^ ^]^ Unlike in our previous studies, where SNCs were further modified with PEGylation, we utilized the positively charged SNCs as a platform to load negatively charged mRNAs, enabling us to study the impact of stiffness on SNC endosomal escape and subsequent mRNA transfection.

### 2.2 Development and Validation of EEA

**Figure 3:**
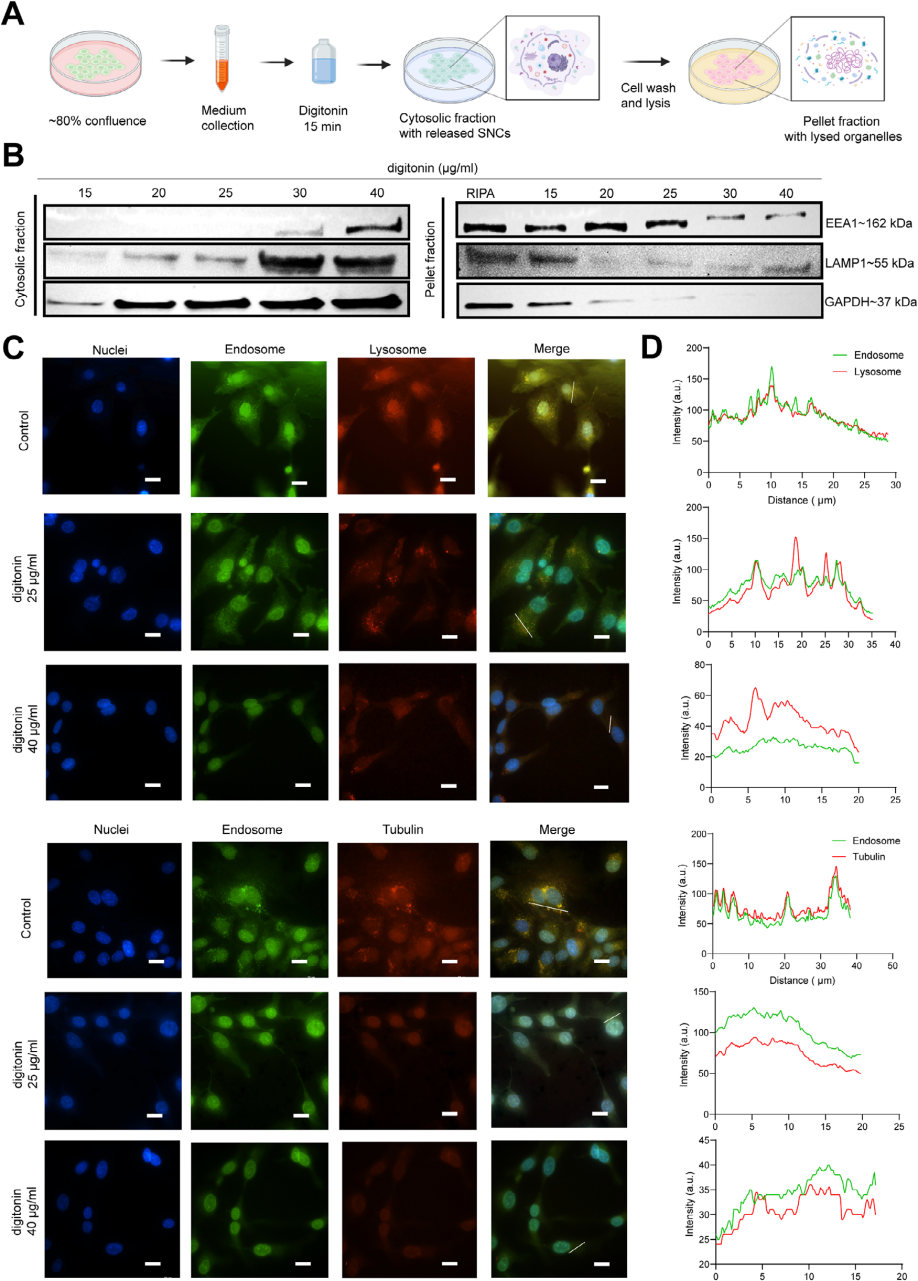
Development and validation of endosomal escape assay. A) Experimental design for separating the cytosol and organelles of SKOV3 cells using digitonin treatment. B) Western blot analysis of cytosolic and organelle markers with various concentrations of digitonin. C) Schematic representation of the effect of semi‐permeabilization on cytosolic content and organelles (scale bars, 20 µm). Cells were stained before digitonin treatment using CellProbe endosome (green), LysoTracker (red) or tubulin (red). The nucleus was stained with Hoechst (blue). D) Intensity profile of endosome‐GFP colocalization with lysosome and tubulin, along the straight line (white, marked on merged images) crossing the cytosol of the representative cell(s).

Quantifying NP endosomal escape within cells is challenging due to the lack of a direct and reproducible assay for comparing different samples. To address this, we developed an EEA based on cell fractionation, which separates cytosolic components from target organelles for quantification (**Figure** **3**A). Digitonin, a non‐ionic detergent, selectively disrupts the plasma membrane, allowing the release of cytosolic components while keeping organelles intact. After incubation with varying concentrations of digitonin (15, 20, 25, 30, and 40 µg mL^−1^) for 15 min on ice, the supernatant was collected as the cytosolic fraction.^[^ ^22^, ^23^, ^24^ ^]^ The components within the organelles extracted using RIPA lysis were referred to as the pellet fraction. An optimized semi‐permeabilization protocol was evaluated by assessing the efflux of cytosolic and organelle markers in the cytosolic and pellet fractions.

Figure 3B shows the western blot analysis of the fractions from SKOV3 cells. The protein levels of the markers in the cytosolic fraction are inversely proportional to those in the pellet fractions. With 25 µg mL^−1^ digitonin treatment, negligible levels of the endosomal marker early endosomal antigen 1 (EEA1) and the lysosomal marker lysosomal‐associated membrane protein 1 (LAMP1) were detected in the cytosolic fractions. In contrast, high levels of the cytosolic protein marker glyceraldehyde 3‐phosphate dehydrogenase (GAPDH) were observed, indicating successful efflux of cytosolic components. Further ELISA analysis showed a similar pattern for each marker, consistent with the band intensities observed on the blots (Figure S1, Supporting Information). These results demonstrate that 25 µg mL^−1^ digitonin treatment in the EEA enables effective separation of cytosolic components from endosome and lysosome organelles.

The distribution of cytosolic and organelle markers was further confirmed by fluorescence microscopy (Figure 3C; Figure S2, Supporting Information). The SKOV3 cells were stained prior to digitonin treatment. The control and low concentration digitonin treatments exhibited typical cell staining patterns, with distinct tubulin throughout the cell structure, and discernible fluorescence in endosomes and lysosomes (Figure 3C; Figure S2, Supporting Information). Treatment with 25 µg mL^−1^ digitonin semi‐permeabilized the cells, resulting in a significant efflux of cytosolic components (indicated by the loss of tubulin signals), while endosomes and lysosomes remained intact. However, incubation with 40 µg mL^−1^ digitonin resulted in the loss of cytosolic and organelle signals, while the nucleus remained intact. Additionally, we observed the strong colocalization of endosome with lysosome/tubulin in the controls (Figure 3D). Nevertheless, following digitonin treatment, both the overlapping and intensity signals decreased, further confirming the separation of cytosolic components.

Fluorescence labelling is a commonly used method to study the intracellular distribution of NPs.^[^ ^25^, ^26^ ^]^ While fluorescence microscopy allows direct visualization of cellular compartments and materials, it only provides semi‐quantitative results. A prior quantification assay called SLEEQ was developed based on the large BiT protein (LgBiT) and its complementary peptide (HiBit).^[^ ^24^ ^]^ However, the assay is time‐consuming, as it relies on the expression of fusion proteins with actin, making it unsuitable for screening studies. In contrast, our EEA offers a cost‐effective and quantitative approach for rapid screening of different NPs. It is important to note that we optimized the EEA in SKOV3 by testing various concentrations of digitonin based on the presence of marker proteins. However, the semi‐permeabilization efficiency using digitonin may vary across different cell lines, and appropriate digitonin concentrations should be determined prior to application.

### 2.3 Effect of NP Stiffness on Cellular Uptake and Cytosolic Delivery

To quantify NP cellular uptake, we labelled SNCs by dissolving Dil in Miglyol 812 oil before forming the SNCs. This approach is advantageous as Dil labels the core of the SNCs for tracking without altering their mechanical properties. First, the correlation between SNC concentrations and fluorescence intensity (FI) was determined in cells used for in vitro EEAs. Cells (2 × 10^4^) seeded in a 96‐well plate were incubated with varying concentrations (from 1 × 10^6^ to 1 × 10^12^ NPs) of SNCs. After the removal of cell medium, FI was measured and normalized against non‐treated controls and background buffers. A linear correlation was established between the NP number from 1 × 10^6^ to 1 × 10^11^ and FI (Figure S3, Supporting Information). The FI readings were either saturated or undetectable when the NP concentration exceeded 1 × 10^11^ NPs or fell below 1 × 10^9^ NPs, respectively. A concentration of ≈1 × 10^10^ NPs provided reliable FI detection while preventing saturation.

**Figure 4:**
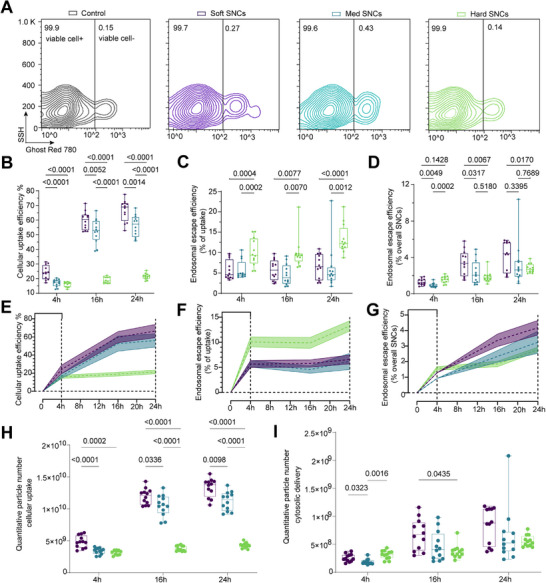
Effect of NP stiffness on cellular uptake and endosomal escape. A) Representative flow cytometry contours showing cytotoxicity of SNCs in SKOV3. B) Cellular uptake efficiency of SNCs at 4, 16 and 24 h. Endosomal escape efficiency of SNCs, calculated by the ratio of cytosolic FI signal to intracellular retention FI (% uptake) (C), and overall associated signal (% overall SNCs) (D). Trends in uptake rates (E) and escape efficiency rates (F‐G) of SNCs at 4, 16 and 24 h. H) Particle number of intracellular SNCs at 4, 16 and 24 h. I) Particle number of cytosolic SNCs at 4, 16 and 24 h. Data represent n = 12 and are displayed as mean ± SD. n = measurement, from 4 independent experiments. Statistical significance was considered at *p* < 0.05. P‐values are determined using two‐way ANOVA followed by Tukey's multiple comparison test.

To determine the optimal NP dose for cellular uptake experiments, we tested three NP numbers, 2 and 4 × 10^10^‐using cell viability and apoptosis assays^[^ ^27^, ^28^ ^]^ (Figures S4 and S5, Supporting Information). Numbers of 1–2 × 10^10^ NPs did not affect cell viability, regardless of their stiffness (**Figure** **4**A; Figure S4, Supporting Information). However, at 4 × 10^10^ NPs, a slight decrease in cell viability was observed (Figure S4, Supporting Information). We then performed a caspase 3/7 apoptosis assay to visualize and quantify apoptotic signals (Figure S5, Supporting Information). Similarly, no apoptotic signals were detected in experiments using 1–2 × 10^10^ NPs (Figure S5, Supporting Information), while cells treated with 4 × 10^10^ NPs showed elevated apoptotic signals. These results confirm that SNC numbers up to 2 × 10^10^ NPs do not affect cell viability or proliferation. Therefore, we used a NP number of 2 × 10^10^ SNCs in subsequent EEA experiments, allowing for clear detection of Dil fluorescence without inducing toxicity.

Next, we applied EEAs to assess the cellular uptake and endosomal escape of the three SNCs with different stiffness using SKOV3 cells (Figures S6 and S7, Supporting Information). After incubation with the SNCs, cells were washed to remove unbound material and subjected to semi‐permeabilization. Signals from cells directly lysed with RIPA buffer served as a baseline for overall intracellular uptake and endosomal escape efficiency, as this method lyses all plasma membranes for FI detection. Background readings, including those from cell medium and buffers, were subtracted from all corresponding measurements.

As expected, the cellular uptake of all three SNCs showed an increasing trend over incubation times (Figure 4B) (*11, 18*). After 4 h, 24.21% of the total soft SNCs had been internalized, while 16.03% of the hard SNCs had been internalized. The maximal cellular uptake efficiency occurred with soft SNCs at 24 h (66.56%), which was 3 times greater than the uptake of hard SNCs (21.45%). It is well established that particle softness is strongly associated with increased cellular internalization.^[^ ^11^, ^12^ ^]^ To better determine the endosomal escape efficiency of these SNCs, we calculated the ratio of cytosolic FI signal to the intracellular retention FI (% uptake) and the overall associated signal (% overall SNCs) (Figure 4C,D).

In the 4 h assay, the endosomal escape efficiency of the hard SNCs was 10.06% (% uptake), which was significantly higher (≈1.8 fold) than that of the med SNCs (5.70%, P = 0.0002) and soft SNCs (5.66%, P = 0.0004). Additionally, the hard SNCs showed the highest endosomal escape efficiency at 4 h, with 1.56% (% overall SNCs) reaching the cytosol. This finding aligns with prior studies on endosomal escape efficiency assessed by other analytical technologies and NP formulations at the 4 h time point.^[^ ^13^, ^24^, ^25^ ^]^ The cellular internalization and endosomal escape rates of SNCs, regardless of stiffness, increased over the time in first 4 h, and then decreased over time (Figure 4E–G), supporting the finding that endosomal escape is a rate‐limiting and inefficient step (*14, 15*).

To further validate the intracellular distribution of SNCs, we quantified the particle number of cellular uptake and cytosolic release (Figure 4H–M). The particle numbers were calculated by converting the corresponding FI and efficiency based on the overall SNCs treatment signals (Figure S3, Supporting Information). The quantitative particle numbers for cellular uptake and endosomal escape showed a similar trend to the uptake efficiency and overall escape efficiency (Figure 4H–M). Notably, at 4 h, hard SNCs showed a superior cytosolic presence with ≈3 × 10^8^, compared to soft SNCs with ≈2.5 × 10^8^ NPs. However, as the incubation time increased, this advantage diminished. Differences in particle stiffness and incubation time may influence the uptake pathways.^[^ ^11^, ^16^, ^17^, ^18^ ^]^

### 2.4 Effect of Particle Stiffness on mRNA Transfection

After evaluating the intracellular distribution of SNCs, we proceeded with in vitro transfection assays using these SNCs. We first attempted to load mRNA‐GFP onto the synthesized SNCs at varying mixing ratios (Figures S8 and S9, Supporting Information). It was expected that the mRNA molecules would bind the positively charged SNCs through electrostatic interactions (Figure S10, Supporting Information). The optimal binding ratio was determined to be 1 NP per 30 mRNA molecules in RNase‐free water, as this ratio resulted in minimal residual mRNA in the supernatant compared to the control, irrespective of SNC stiffness (Figure S10, Supporting Information, P < 0.0001, ≈0.1 fold change). A noticeable decrease in zeta potential (SNCs‐mRNA suspended in RNase‐free water with ionic strength changes) was observed across all SNCs groups (Figure S10, Supporting Information), indicating successful absorption of mRNAs onto the SNCs.

**Figure 5:**
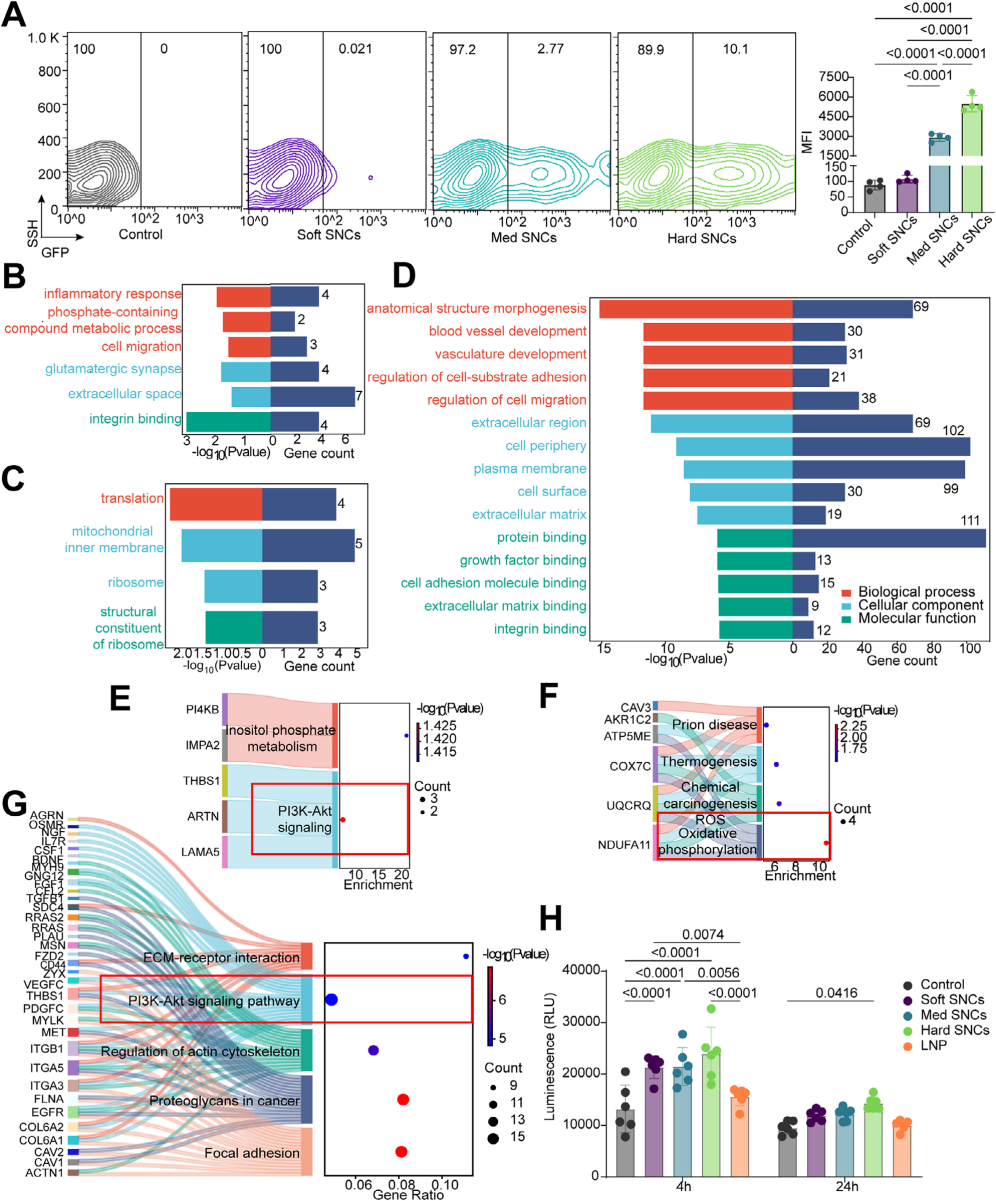
Effect of NP stiffness on transfection and cell signaling. A) Representative flow cytometry contours of transfection efficiency (%) of SNCs‐mRNA (left) and mean fluorescence intensity (MFI) of cells treated with SNCs‐mRNA (right). Data represent n = 4 and are displayed as mean ± SD. n = biological replicates, each performed in technical triplicate. Statistical significance and P‐values are determined using one‐way ANOVA followed by Tukey's multiple comparison test. Enriched GO analysis of DEGs following soft (B), hard (C) SNC treatments and pooled LIPO/PLGA/PS (D) treatment. Enriched KEGG pathway identified from DEGs following soft (E), hard (F) SNC treatment and pooled LIPO/PLGA/PS (G) treatment. The pooled LIPO/PLGA/PS include multiple formulations of liposomes (LIPO), polylactide‐co‐glycolide (PLGA) and polystyrene (PS) NPs. Significantly enriched terms are displayed (P < 0.05). H) Levels of ROS generation at 4 and 24 h after treatment with SNCs and LNPs, with untreated cells as the control. Data represent n = 6, and results are displayed as mean ± SD. n = measurement, from 3 independent experiments. Statistical significance was considered at *p* < 0.05. P‐values are determined using two‐way ANOVA followed by Sidak's multiple comparison test.

After 4 h of SNCs incubation (followed by an additional 20 h incubation with fresh medium), the hard SNCs demonstrated enhanced mRNA delivery, as indicated by GFP expression (Figure S10, Supporting Information). No GFP fluorescence signal was observed for the mRNA‐loaded soft SNCs. These findings align with the EEA results that stiff SNCs facilitate rapid cytosolic delivery within 4 h. Subsequent flow cytometry analysis further confirmed significant GFP expression (10.1% transfection efficiency and MFI = 5488) in cells treated with hard SNCs‐mRNA (**Figure** **5**A, P < 0.0001 compared to the control and softer SNCs). Interestingly, increasing the SNCs‐mRNA incubation time to 24 h (Figure S10, Supporting Information) showed a similar expression pattern, with hard SNCs continuing to exhibit greater transfection efficiency over soft and med SNCs. This may be due to the superior capability of hard SNCs in transporting mRNA than softer counterparts.

### 2.5 NP Stiffness‐Associated Cell Response in Cytosolic Delivery

Next, we applied RNA sequencing to investigate the impact of NP stiffness on cellular signaling pathways. To determine the optimal sequencing time point, we quantified the expression of common inflammatory genes (CXCL2 and TNF) after 4, 24, and 48 h NP exposure^[^ ^29^ ^]^ (Figure S11, Supporting Information). Our results revealed that 4 h is a critical time point for inducing cellular responses (e.g., stress response genes), which is consistent with previous studies.^[^ ^13^, ^14^, ^15^, ^24^, ^25^, ^29^ ^]^ Based on these findings, RNA was extracted after 4 h of SNCs incubation and subjected to sequencing (Figure S11, Supporting Information).

We then performed differential expression (DE) analysis to compare the transcriptomes of cells treated with different SNCs (control vs soft SNCs, control vs med SNCs, and control vs hard SNCs). First, we pooled the samples together to identify common cellular responses after SNC treatment. Principal component analysis (PCA) revealed that SNC treatment led to transcriptomic changes, accounting for ≈50% of the total variance in treatment responses (Figure S12, Supporting Information). The resulting volcano plots provided an overview of the differentially expressed genes (DEGs) (Figure S13, Supporting Information),^[^ ^30^ ^]^ identifying a total of 397 genes significantly associated with all three SNCs treatment (Figure S14, Supporting Information). When mapped to the DAVID database,^[^ ^31^ ^]^ the top three significantly enriched Kyoto Encyclopedia of Genes and Genomes (KEGG) pathways were “ribosome”, “coronavirus disease‐COVID‐19” and “Alzheimer's disease” (Figure S14, Supporting Information). Previous studies have shown that exposure to silica NPs involves pathways related to ribosome and Alzheimer's disease.^[^ ^32^, ^33^ ^]^ In this work, we are the first to identify an association between COVID‐19 signaling and SNCs with tunable stiffness, suggesting that these SNCs may trigger a cellular response similar to that of COVID‐19 infection.

The enriched KEGG genes (Figure S14, Supporting Information) were entered into the Retrieval of Interacting Genes/Proteins (STRING) database to generate a highly interconnected protein‐protein interaction (PPI) network (Figure S14, Supporting Information, 30 nodes and 258 edges).^[^ ^34^ ^]^ Further Gene Ontology (GO) analysis showed that these DEGs were closely associated with cytosol and ribosomal activities^[^ ^35^ ^]^ (Figure S14, Supporting Information).

A Venne diagram uncovers 54 and 60 DEGs that are unique to soft and hard SNC treatments, respectively (Figure S14, Supporting Information). However, no pathways specifically associated with med SNCs were identified, likely because the gene set (46 genes) was not significantly enriched in any particular signaling pathway (Figure S14, Supporting Information).

To determine stiffness‐dependent cellular responses, we analyzed DEGs from the soft and hard SNC groups and compared them with a previous study using pooled NPs, including multiple formulations of liposomes (LIPO), polylactide‐co‐glycolide (PLGA), and polystyrene (PS)^[^ ^34^ ^]^ (Figure 5). The DEGs associated with soft SNCs were enriched in GO terms related to cell migration (BP), extracellular space/region (CC), and integrin binding (MF) (Figure 5B). Additionally, the PI3K‐Akt signaling pathway was closely linked to soft SNCs (Figure 5E). Interestingly, similar cellular associations (GO terms and KEGG pathways) were observed in pooled multiple nanoformations of LIPO/PLGA/PS NPs (Figure 5D,G)^[^ ^34^, ^36^, ^37^, ^38^, ^39^, ^40^, ^41^ ^]^. In contrast, the DEGs identified in hard SNCs (high stiffness model, GPa) were significantly enriched in GO terms related to translation, mitochondrial membrane, and ribosomes (Figure 5C). The ROS/oxidative phosphorylation pathway was notably altered in responses to hard SNCs (Figure 5F), highlighting how NP stiffness can significantly influence intracellular response and signaling.

To further validate the impact of oxidative stress induced by hard SNCs on cellular uptake and endosomal escape, we examined ROS generation following SNC treatment. To eliminate constraints associated with silica nanoformulations, Moderna LNPs were used for comparisons (Table S2, Supporting Information; Figure 5H). The 4h‐ROS assay showed that hard SNCs generated the highest ROS levels, with luminescence signals twice those of the control (P < 0.0001) and LNP (P < 0.0001). After an additional 20 h incubation with fresh medium (24 h assay), the hard SNCs continued to exhibit the strongest ROS signals, while no significant differences were observed in the treatment groups of soft SNCs, med SNCs and LNP (Figure 5H). These results suggest that NP stiffness‐mediated ROS may play a key role in regulating NP intracellular delivery and endosomal escape.^[^ ^42^ ^]^

### 2.6 Role of Stiffness‐Induced ROS in Mitochondria and NP Transfection

**Figure 6:**
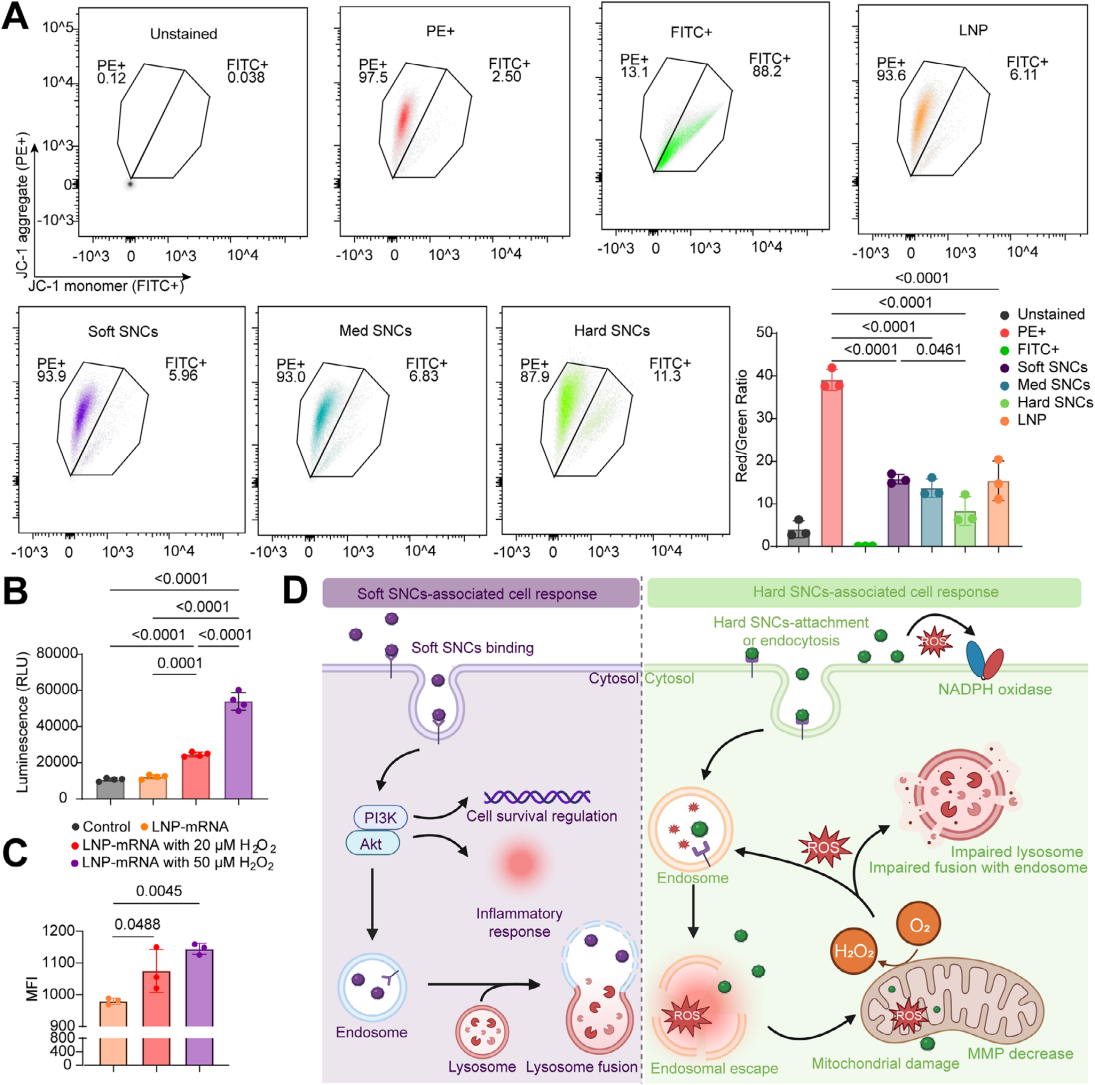
Effect of stiffness‐induced ROS in mitochondria and NP transfection. A) Flow cytometry dot plots showing JC‐1 aggregate‐area (PE+) versus JC‐1 monomer area (FITC+) and corresponding fluorescence intensity histograms of cells with JC‐1 monomers (FITC+). Carbonyl cyanide chlorophenylhydrazone (CCCP, a mitochondria disruptor) was used as a control (FITC+). MMP was calculated as the red (PE+)/green (FITC+) cell population ratio, indicating depolarization/damage of the mitochondrial membrane. Data represent n = 3 and are displayed as mean ± SD. n = biological replicates, each performed in technical triplicate. B) ROS generation level at 4 h after treatment with LNP‐mRNA with and without H_2_O_2_. Data represent n = 4 and are displayed as mean ± SD. n = biological replicates, each performed in technical triplicate. C) Flow cytometry analysis showing MFI of cells co‐treated with LNPs‐mRNA and H_2_O_2_. Data represent n = 3 and are displayed as mean ± SD. n = biological replicates, each performed in technical triplicate. D) Potential cellular response shift based on particle stiffness: soft SNCs (left) bind to receptors, altering the PI3K‐Akt pathway, leading to cellular phenotypes such as cell proliferation and inflammatory responses.^[^ ^39^ ^]^ In contrast, hard SNCs (right) trigger greater ROS production and mitochondrial dysfunction.^[^ ^37^, ^48^ ^]^ The induced oxidative stress not only accelerates cargo release but also impairs lysosomal function, preventing fusion with endosomes.^[^ ^49^ ^]^ Results are displayed as mean±SD. Statistical significance was considered at *p* < 0.05. P‐values are determined using one‐way ANOVA followed by Tukey's multiple comparison test. Part of the figure was created with BioRender.com.

Next, we sought to determine the mitochondrial membrane potential (MMP), which is associated with ROS generation and oxidative stress.^[^ ^43^, ^44^, ^45^ ^]^ MMP was measured using the JC‐1 detection assay, where cells treated with the three SNCs and LNP for 4 h were subjected to flow cytometry analysis (**Figure** **6**A).^[^ ^46^ ^]^ Cells exposed to hard SNCs exhibited the lowest MMP (significant green shift) in the 4 h assay, with this effect being more pronounced in the 24 h assay (Figure S15, Supporting Information). These findings further support the notion that NP stiffness induces distinct intracellular responses by altering MMP and ROS levels, consistent with the results from RNA sequencing, ROS assays, and previous studies.^[^ ^42^, ^47^ ^]^

Therefore, it is likely that the ROS response serves as a positive regulator for intracellular delivery and transfection efficiency. To test this hypothesis, we examined the role of ROS and oxidative stress using a commercial LNPs system. SKOV3 cells were co‐cultured with LNP‐mRNA and low levels of H_2_O_2_ for 4 h. As expected, significant ROS production (P < 0.0001) was observed in the H_2_O_2_‐treated groups compared to the untreated control and LNPs‐mRNA group (Figure 6B). Enhanced GFP expression (MFI = 1075 and 1144 compared to LNP‐mRNA alone MFI = 979.7) and transfection efficiency (24.8% and 33.9% compared to LNP‐mRNA alone, 15.5%) were also observed in cells co‐treated with LNP‐mRNA and H_2_O_2_ (Figure 6C; Figure S16, Supporting Information). However, increased incubation time (>8 h) or higher H_2_O_2_ concentrations (100 µm) induced cell death, as evidenced by morphological changes and cell detachment (data not shown). These results suggest that low levels of ROS‐mediated oxidative stress boost NP delivery and transfection.

## 3 Discussion

In this work, we synthesized three types of SNCs with tunable stiffness to investigate the impact of NP stiffness on intracellular distribution profile, cellular uptake level, and endosomal escape efficiency. We developed an EEA, enabling the quantitative assessment and comparison of NP cellular uptake and endosomal escape capabilities. This assay can be applied to different NPs and cell types for comparative studies. RNA‐seq analysis identified distinct signaling pathways associated with NP stiffness that enhance endosomal escape.

Based on the EEA, we demonstrated that while the soft SNCs exhibited greater uptake efficiency (24.21%), the hard SNCs showed a 1.8 fold enhancement in endosomal escape efficiency (10.06%, % uptake) within 4 h in cancer cells. In our previous studies, hard SNCs showed a 3 fold increase in uptake by macrophages compared to soft SNCs, although no significant uptake difference was observed in cancer cells. Additionally, hard SNCs functionalized with targeting ligands, such a folic acid, displayed significantly higher uptake in cancer cells.^[^ ^21^ ^]^ The differences in cellular uptake are mainly due to the distinct surface properties of SNCs between our previous and current studies. In the previous work, SNCs were PEGylated, resulting in a slightly negatively charged system,^[^ ^5^ ^]^ whereas in the current study, they are positively charged to serve as a platform for loading negatively charged mRNAs for transfection studies. Positively charged NPs are more effectively taken up by cells (especially macrophages) due to favorable electrostatic interactions with the negatively charged cell membrane, whereas PEGylation can inhibit NP uptake.^[^ ^5^ ^]^

Taking advantage of the positive charge of SNCs, mRNA was loaded through electrostatic absorption. At both 4 and 24 h treatment, mRNA‐loaded hard SNCs showed significantly greater transfection efficiency (10.1%) compared to their softer counterparts (0.2% for soft SNCs and 2.7% for med SNCs), indicating a strong association between NP stiffness and transfection efficiency. Given the GFP expression level observed in this study, electrostatic interactions can be easily utilized to load RNA cargo onto positively charged SNCs. However, an important consideration is to maintain the stability of genetic materials for efficient transfection. The layer‐by‐layer (LbL) method, which uses oppositely charged polymers to form an outer shell, has been successfully applied to various NPs to protect naked nucleic acids, resulting in improved uptake and controlled release.^[^ ^50^, ^51^, ^52^ ^]^ For example, biocompatible hyaluronic acid (HA) has been used as an outer layer for NPs to improve uptake and targeted delivery.^[^ ^52^, ^53^ ^]^ Future efforts could leverage the LbL assembly approach to stabilize the SNCs‐mRNA complex for enhanced transfection efficiency.

Using RNA‐seq analysis, we identified distinct signaling pathways associated with NP stiffness (Figure 6D). Soft SNCs regulate intracellular delivery via the PI3K‐Akt pathway, while hard SNCs activate the reactive oxygen species (ROS)‐mediated mechanism. Interestingly, similar pathways were observed in the pooled NPs in literature, including LIPO and PLGA NPs coated with a series of polyanions, as well as PS NPs modified with carboxylate and sulfate groups.^[^ ^34^ ^]^ In contrast, hard SNCs specifically induced ROS‐mediated signaling. Although ROS production has been implicated in nanotoxicity (e.g., from positive surface charges) ^[^ ^54^, ^55^ ^],^ its role in NP uptake and escape has not been thoroughly explored. We validated stiffness‐induced oxidative stress as a regulator of NP uptake and endosomal escape, demonstrating that our SNCs at normal concentrations are non‐toxic (Figure 4A; Figures S4 and S5, Supporting Information). Furthermore, some degree of ROS production was observed across all NPs, with positively charged SNCs inducing a greater ROS response over PEGylated SNCs and LNPs, both of which are slightly negatively charged (Table S3 and Figure S17, Supporting Information). Interestingly, NPs that induce ROS accumulation have been shown to activate immune responses by boosting CD8^+^ T cell infiltration, reducing M2 macrophage presence, and promoting cancer cell death, thereby improving the immunosuppressive microenvironment.^[^ ^56^, ^57^ ^]^ However, given the variability in ROS sensitivity and its biological effects across different cell populations, it is critical to establish precise control for the safe use of ROS.^[^ ^58^ ^]^ Building on previous findings, there may be an “optimal” ROS response and level of oxidative stress that enhances cytosolic delivery while balancing the trade‐off between nanotoxicity and delivery efficiency.

Different stiffness levels of NPs may activate distinct cellular binding and internalization pathways. For example, the shape transformation has been observed in various soft NPs.^[^ ^18^, ^20^, ^59^ ^]^ In HeLa cells, stiff LNPs (1.2 GPa) had a better uptake efficiency compared to softer LNPs (0.76 GPa).^[^ ^59^ ^]^ The dynamic simulations suggest that softer LNPs undergo the morphological change, which requires more energy and thus limits the uptake capacity. However, the stiff LNPs are believed to be easily engulfed by cancer cells. In our previous study, PEGylated soft and hard SNCs show similar non‐specific binding and uptake efficiency in SKOV3 cells, as their endocytic pathway is independent of clathrin, caveolin, or F‐actin polymerization.^[^ ^18^ ^]^ Interestingly, folic acid‐PEG‐modified hard SNCs exhibited significantly higher uptake in SKOV3 through receptor‐mediated endocytosis, which indicates a synergistic effect between surface conjugation and NP stiffness. In another study, PEG‐modified soft silica NPs (47 MPa) displayed a higher cellular uptake in MCF‐7 cells than their hard counterparts.^[^ ^20^ ^]^ These inconsistencies may be contributed by different nanomaterials, surface modifications, and in vitro disease models.

Overall, in this study, we tuned the SNCs stiffness by mixing two different silica precursors at varying TEVS/TEOS molar ratios. We showed that these SNC formulations, with stiffness ranging from 1.37 MPa to 1.72 GPa, spanning orders of magnitude, maintain similar physical properties (size:175 ± 10 nm; PDI < 0.15, and ζ potential: 40 ± 5 mV). The variation solely in mechanical properties provides an ideal model system for exploring the impact of NP stiffness on internalization and biological responses. In addition, taking advantage of optimized EEA, it was found that soft SNCs exhibited 3 fold enhanced cellular uptake (66.56% internalized soft SNCs versus 21.45% internalized hard SNCs) after 24 h incubation with human ovarian cancer SKOV3 cells. Notably, we first observed that hard SNCs demonstrated superior endosomal escape efficiency (≈1.8 fold) in the 4 h assay, with 10.06% escaping versus 5.70% for med SNCs and 5.66% for soft SNCs. The sequencing then revealed distinct cellular responses mediated by NP stiffness. The hard SNCs induce the reactive oxygen species (ROS)‐mediated oxidative stress, which enhances the endosomal escape and cytosolic release of NPs. In contrast, soft SNCs activate the PI3K‐Akt pathway, which is involved in cell proliferation and inflammatory responses. This is the first work to investigate NP stiffness in cellular uptake, endosomal escape, and potential biological relevance, which not only provides new and valuable insights into how NP stiffness regulates intracellular distribution but also offers guidance for the design of NPs to enhance endosomal escape and improve cytosolic delivery.

## 4 Experimental Section

### Preparation and Characterization of SNCs

### Preparation and Characterization of SNCs—Synthesis of Nano‐Emulsion and SNCs with Tunable Stiffness

The bifunctional peptide SurSi: Ac‐MKQLAHSVSRLEHARKKRKKRKKRKKGGGY‐CONH_2_ (MW 3632, with a purity ≥ 95%) was custom‐synthesized by GenScript Corporation (Piscataway, NJ, USA). The Sursi peptide was dissolved in 25 mm HEPES buffer (pH 7.5) at a concentration of 400 µm, supplemented with 800 µm of ZnCl_2_. To the peptide solutions, 2% v/v of Miglyol 812 oil (Caesar & Loretz GmbH, Hilden, Germany) was added, followed by sonication at 10–13% amplitude for five 30 s bursts with 60 s intervals on ice. The diameter and zeta potential of the nano‐emulsions were measured using dynamic light scattering (DLS, Malvern Zetasizer Ultra, Malvern Instruments, UK).

The resulting nano‐emulsions were used to synthesize SNCs with tunable stiffness, as described in a previous study.^[^ ^18^ ^]^ Briefly, to synthesize soft and med SNCs, a 25 mm HEPES buffer at pH 7.5 was mixed with the nano‐emulsion at a 1:1 volume ratio, followed by incubation with a 160 mm solution of TEOS/TEVS at a ratio of 1:1. The reaction was performed with magnetic stirring at 500 rpm at 28 °C for 24 h, followed by dialysis using a 10 kDa molecular weight cutoff membrane (Thermo Fisher Scientific, USA) in 2.5 mm HEPES at pH 7.5 for 20 h in a cold room. For hard SNC synthesis, the nano‐emulsion was first dialyzed and then mixed at a 1:1 ratio with 2.5 mm HEPES buffer at pH 7.5. A 160 mm solution of TEOS/TEVS was then added, with TEOS making up 90% of the molar content. The reaction was performed with incubator shaking at 180 rpm at 28 °C for 24 h, followed by further dialysis using the procedure described above.

The hydrodynamic particle size, zeta‐potential, and particle concentration of the SNCs were characterized using DLS. Particle morphology was examined by TEM using an FEI Tecnai G2 Spirit TEM operated at 120 kV (FEI, US). The mechanical properties of SNCs were determined using a Dimension Icon XR AFM (Bruker, Billerica, MA, USA) following the procedures described previously.^[^ ^18^ ^]^ The SNCs were dialyzed against Milli‐Q water at 4 °C for 20 h to remove salts. A 20 µL of SNCs was placed onto the cleaved mica surface with air dry. The soft and med SNCs were scanned in water using a DNP‐10 cantilever with a spring constant of 0.24 N/m, while the hard SNCs were scanned in air using a RTESPA‐150 cantilever with a spring constant of 5 N/m. For each sample, 5 different SNCs were analyzed and the Young's moduli of the SNCs were obtained by fitting the force‐indentation curves using the Hertzian contact model. AFM data were processed using NanoScope Analysis software (v. 2.0).

### PEGylation of SNCs

The surface of the SNCs was modified with PEG as previously described.^[^ ^18^ ^]^ Briefly, 4 mm of 3‐aminopropyltriethoxysilane (APTES) was added to the pre‐synthesized SNCs with magnetic stirring at room temperature for 2 h. Then, 8 mm of mPEG‐NHS, dissolved in 25 mm HEPES buffer (pH 7.2) was added to the mixture for further reaction at room temperature for 4 h. The modified SNCs were then collected and resuspended in 1 × PBS for characterization.

### Preparation and Characterization of LNPs

LNPs were prepared based on the Moderna formulation. Briefly, the components were dissolved in absolute ethanol and mixed in the desired molar ratio (SM‐102:DSPC:cholesterol:DMG‐PEG_2000_ at a molar ratio of 100:20:77:3). Separately, 5 µg of mRNA was dissolved in RNase‐free sodium citrate buffer (100 mm, pH 4). The aqueous and organic solutions were then quickly mixed in a volume ratio of 3:1. The resulting LNPs were subjected to dialysis using a 10 kDa membrane against 1× PBS overnight in a cold room. The dialyzed LNPs were then collected for characterization.

### Cell Culture

SKOV3 cells (HTB‐77, ATCC) were cultured in growth medium containing high‐glucose DMEM (Gibco), 10% fetal bovine serum (FBS, Gibco), and 1% Penicillin‐Streptomycin (Gibco) at 37 °C with 5% CO_2_ until reaching 70%–80% confluence for experiments.

### Separation of Cytosolic Fraction and Organelles by Digitonin

To separate the cytosolic fraction, protocols were optimized based on prior methodology.^[^ ^23^ ^]^ SKOV3 cells were seeded in a 24‐well plate at 6 × 10^4^ cells/well and grown to 80% confluence. The optimal separation protocol was developed by monitoring the release of cytosolic marker (GAPDH), endosomal marker (EEA1), and lysosome marker (LAMP1) from cells treated with various concentrations of digitonin (Sigma–Aldrich) in a solution containing 2 mm DTT and 2 mm MgCl_2_ in 1× NEH buffer (150 mm NaCl, 0.2 mm EDTA and 20 mm HEPES‐NaOH at pH 7.4). Cells were incubated with digitonin on ice with a shaking platform at ∼65 rpm for 15 min. After digitonin treatment, the supernatant was collected as the cytosolic fraction. Cells were then lysed with RIPA lysis buffer (Thermo Fisher Scientific) on ice for 10 min with shaking at ≈65 rpm, followed by centrifugation at 15000 × g for 15 min. The supernatant was collected as the pellet fraction (within organelles). A final working concentration of digitonin (25 µg mL^−1^) was applied to selectively semi‐permeabilize the plasma membrane to separate cytosolic and pellet fractions. Cells directly treated with RIPA lysis buffer served as a control. To prevent protein degradation, 10 µL mL^−1^ of protease and phosphatase inhibitor cocktail (Thermo Fisher Scientific) was added to the buffers

### Localization of Cell Compartments

Following the manufacturers’ instructions, cells were stained with Hoechst 33342 (Invitrogen), CellLight Early Endosomes‐GFP (Thermo Fisher Scientific) and LysoTracker Red DND‐99 (Thermo Fisher Scientific) or CellLight Tubulin‐RFP (Thermo Fisher Scientific), followed by digitonin treatment for 15 min on ice. Cells were then washed twice by DPBS and fixed with 5% paraformaldehyde (PFA, Sigma) for 15 min. Cells were imaged and photographed for localization of cell compartments using a fluorescent microscope (FITC filter, Texas Red filter, DAPI filter, Nikon ECLIPSE Ti2 inverted microscope, Japan). ImageJ (v1.54p) was used to quantify the colocalization between the endosome fluorescence and the lysosome fluorescence.

### Analysis of Protein Samples by SDS‐PAGE Electrophoresis and/or Qubit

To assess the protein content from the cytosolic and pellet fractions, protein samples (10–20 µL) were mixed with laemmli sample buffer and boiled at 95 °C for 5 min before being loaded onto 4–20% Mini‐PROTEAN TGX precast protein gels (Bio‐Rad Laboratories) as described.^[^ ^60^ ^]^ Precision Plus protein dual color ladder (Bio‐Rad Laboratories) was used as a molecular weight marker. Electrophoresis was conducted at a constant voltage of 80 V for 15 min, followed by 120 V for ≈40 min with 1× Tris/Glycine/SDS running buffer (Bio‐Rad Laboratories). After electrophoresis, the gel was stained with SimplyBlue SafeStain (Thermo Fisher Scientific) overnight at room temperature. The gel was washed with water for 1 h, and protein bands were visualized using the iBright CL1500 Imaging System (Thermo Fisher Scientific). Protein expression levels were analyzed using iBright analysis software (Thermo Fisher Scientific). Alternatively, protein samples were quantified using the Invitrogen Qubit Protein BR assay kit (Thermo Fisher Scientific) according to the manufacturer's instructions.

### Western Blot

Cellular protein was subjected to western blot analysis following SDS‐PAGE electrophoresis. Proteins were transferred to 0.2 µm nitrocellulose membranes (Bio‐Rad) using the Trans‐Blot Turbo Transfer System (Bio‐Rad). The membranes were blocked with EveryBlot blocking buffer (Bio‐Rad) at room temperature for 15 mins, followed by incubation with specific primary antibodies overnight at 4 °C: anti‐GAPDH antibody (ab9485, Abcam, Australia), anti‐EEA1 antibody (ab109110, Abcam, Australia), and anti‐LAMP1 antibody (LAMP1‐101AP, Thermo Fisher Scientific, Australia). After rinsing with 1× TBST, the membranes were incubated with a goat anti‐rabbit IgG (H + L)‐HRP conjugate secondary antibody (170‐6515, Bio‐Rad) solution for 1 h at room temperature. The blots were then washed with 1× TBST (Thermo Fisher Scientific), and immunodetection was visualized using the iBright CL1500 Imaging System (Thermo Fisher Scientific). Protein expression levels were analyzed using iBright analysis software (Thermo Fisher Scientific).

### ELISA

The Human GAPDH ELISA Kit (Invitrogen), Human LAMP1/CD107a ELISA Kit (Invitrogen), and Human Early Endosome Antigen 1 (EEA1) ELISA Kit (MyBioSource) were used to quantify the levels of target proteins in the samples. Standards were serially diluted, and assay solutions were prepared following the manufacturers’ protocols. GAPDH, LAMP1, and EEA1 concentrations were measured by adding 100 µL of total protein to the ELISA plate, followed by absorbance reading at 450 nm. The absorbance reading from the blank was subtracted from the readings of standards, controls, and samples.

### Cell Viability and Proliferation Assay

Cells were cultured in growth medium until they reached 80% confluence. To examine the effect of SNC treatment on cell viability and proliferation, cells were treated with various concentrations of SNCs (1, 2, and 4 × 10^10^) for 24 h. For the WST assay, cells were treated with a medium containing 10% WST reagent (Sigma) and incubated for an additional 2 h. Plates were gently shaken before absorbance was measured at 450 nm using a TECAN Infinite 200 PRO plate reader. For the Ghost Dye viability assay (Cytek Biosciences), cells were dissociated with TrypLE express enzyme (Gibco) and resuspended in 1 × PBS (Sigma) supplemented with 2% FBS prior to Ghost Dye staining (1:1000 dilution) for 30 min on ice. Samples were then analyzed using the BD FACSVia flow cytometry (PE filter, US), and data were processed using FlowJo software (v10.10.0). Wells containing only medium were used as blank controls, while wells with cells but without SNC treatment served as positive controls.

### Cell Apoptosis Assay

Cells (2 × 10^4^) were cultured in growth medium until 80% confluence and treated with SNCs (at 1, 2, 3 × 10^10^ NPs) for 24 h. The cells were then incubated with 80 µL of CellEvent Caspase‑3/7 green reagent (Thermo Fisher Scientific) for 30 min. After incubation, imaging was performed using a fluorescent microscope (FITC/GFP filter, Nikon ECLIPSE Ti2 inverted microscope, Japan). Following imaging, cells were dissociated with TrypLE express enzyme (Gibco) and washed twice with 1× PBS (Sigma) supplemented with 2% FBS. After the final suspension, samples were analyzed using the BD FACSVia flow cytometry (FITC filter, US), and data were processed using FlowJo software (v10.10.0).

### Reactive Oxygen Species (ROS) Assay

The generation of ROS was detected using the luminescence‐based ROS‐Glo H_2_O_2_ assay (Promega). Following the manufacturer's instructions, cells (2 × 10^4^) were cultured in growth medium until 80% confluence in 96‐well plates. The growth medium was then replaced with 100 µL of medium containing 2 × 10^10^ NPs with or without H_2_O_2_ and 25 µm H_2_O_2_ substrate for 4 h. The cells were subjected to the ROS assay for the 4 h time point. For the 24 h assay, cells were first incubated with NPs for 4 h, followed by replacing the medium with 80 µL of fresh medium for an additional 20 h of incubation. The cells were then supplemented with 20 µL of 25 µm H_2_O_2_ substrate for the final 4 h. Next, 100 µL of the prepared ROS‐Glo detection solution was added to the wells and incubated for 20 min at room temperature. Luminescence signals were measured using a TECAN Infinite 200 PRO plate reader. Cells without NP treatment served as untreated control. Subtract‐only normalization applied.

### Mitochondrial Membrane Potential (MMP) Assay

Cells (6 × 10^4^) were cultured in growth medium until 80% confluence and treated with SNCs and LNPs (6 × 10^10^) for 4 h. The cells were either subjected to the MMP assay immediately or further incubated with fresh medium for an additional 16 h (24 h assay). The harvested cells were resuspended in 1× PBS, and the MMP assay was performed using the MitoProbe JC‐1 assay kit (Thermo Fisher Scientific). Briefly, cells were incubated with 2 µm of JC‐1 probe for 20 min at 37 °C, followed by two washes with 1× PBS supplemented with 2% FBS. After the final suspension, samples were analyzed using the BD FACSVia flow cytometry (JC‐1 aggregate‐PE filter, JC‐1 monomer‐FITC filter, US), and data were processed using FlowJo software (v10.10.0).

### Quantification of SNC Intracellular Distribution

In order to quantify the intracellular distribution of SNCs, cells were seeded into 96‐well plates at a density of 10^4^ cells per well and incubated overnight. The following day, cells were treated with SNCs (2 × 10^10^ NPs) and harvested at 4, 16, and 24 h time points. The treatment medium was collected and transferred to a 96‐well black plate for fluorescent intensity detection. Each well was washed with 20 µL of cold DPBS (Thermo Fisher Scientific) and the wash was transferred to corresponding wells labeled as efflux fractions. Cells were subsequently treated with 100 µL of 25 µg mL^−1^ digitonin on ice for 10 min with shaking at ≈65 rpm. The supernatant was collected and transferred to a 96‐well black plate. Each well was washed twice with 50 µL of cold DPBS, and the washes were transferred to wells labeled as cytosolic fractions. To extract the pellet, 100 µL of RIPA lysis buffer (Thermo Fisher Scientific) was added to each well and incubated on ice for 10 min with shaking at ≈65 rpm. The lysate was transferred to a 96‐well black‐bottom plate, and each well was washed twice with 50 µL of cold DPBS, with the washes transferred to wells labeled as pellet fractions. Fluorescence readings (Dil λex = 520 nm and λem = 565 nm) were measured using a TECAN Infinite 200 PRO plate reader.

### Biotherapeutic Binding of SNCs

The mRNA encoding the fluorescent protein reporter eGFP was obtained from the BASE mRNA facility at The University of Queensland, Australia. The binding ratios of NPs to mRNA were tested at 1:10, 1:20, 1:30, and 1:40. After preliminary tests, a binding ratio of 1:30 (1 NP binding with 30 mRNA molecules) was established. mRNA was loaded onto the positively charged surfaces of the SNCs by gently mixing the SNC suspension with the corresponding amount of mRNA with a pipette, followed by incubation for 15 min at room temperature. The mixture was then centrifuged at 20 000 × g for 10 min, and the supernatant, containing the remaining unbound mRNA, was collected for RT‐PCR analysis to quantify the mRNA bound to SNCs. The pellet was resuspended in RNase‐free water for DLS characterization.

### Polymerase Chain Reaction (PCR)

Reverse transcription‐polymerase chain reaction (RT‐PCR) analysis was carried out to measure the residual mRNA after binding to SNCs. The collected supernatant containing unbound mRNA was subjected to reverse transcription, with mRNA only used as a control. First‐strand complementary DNA (cDNA) was reverse‐transcribed from the collected supernatant samples using the iScript cDNA synthesis kit (Bio‐Rad).

Specific primers (Table S1, Supporting Information) were designed using NCBI Primer Blast and Primer 3.0 InPut software, and synthesized by Integrated DNA Technologies (IDT, Australia). Quantitative PCR was performed using PowerTrack SYBR Green Master Mix (Thermo Fisher Scientific) on a Bio‐Rad CFX PCR system. The relative expression of genes of interest was calculated using the comparative 2^−ΔCt^ method, normalized to the expression of the control gene.

### Biotherapeutics Transfection Efficiency

To examine the transfection efficiency of biotherapeutics, cells at 80% confluence were treated with mRNA‐loaded SNCs (2 × 10^10^ SNCs with 6 × 10^11^ mRNA molecules) for 4 and 24 h. For fluorescent imaging, cells were fixed with 5% paraformaldehyde (PFA, Sigma) for 15 min and imaged using a fluorescent microscope (mRNA‐FITC/GFP filter, Nikon ECLIPSE Ti2 inverted microscope, Japan). For flow cytometry analysis, cells were harvested and suspended in 1 x PBS (Sigma) supplemented with 2% FBS. After final suspension, samples were analyzed using the BD FACSVia flow cytometry (mRNA‐FITC/GFP filter, DNA‐PE filter, US), and data were processed using FlowJo software (v10.10.0).

### RNA Extraction

Total RNA was extracted using the PureLink RNA Mini Kit (Thermo Fisher Scientific) following the manufacturer's instructions. The purity of the extracted RNA was assessed by NanoPhotometer NP80 (IMPLEN, Germany), with an A260/280 ratio of ∼2.0 considered indicative of purified RNA. For quantification, the purified RNA was subjected to the Qubit RNA BR (Broad‐Range) Assay (Invitrogen), following the manufacturer's protocols. The Qubit RNA IQ Assay (Invitrogen) was then performed to determine the integrity and quality of the RNA samples, according to the manufacturer's instructions. A Qubit 4 fluorometer (Thermo Fisher Scientific) was used for all sample readings. An RNA integrity number or quality score ≥ 8 was considered high quality. High‐quality RNA was treated with DNase using the DNA‐free Kit (Invitrogen), and the final DNA‐free RNA samples were submitted for mRNA sequencing.

### mRNA Sequencing

Three replications for each sample (a total of 12, including soft SNCs, med SNCs, hard SNCs, and control) were subjected to mRNA sequencing. The sequencing was performed by the Australian Genome Research Facility (AGRF, SA, Australia).

### RNA‐Seq Analysis

FASTQ files were paired, trimmed, and aligned the using *RNAlysis* (pypi, v3.12.0).^[^ ^61^ ^]^ Differential gene expression (DEG) was determined using DESeq2, with data filtered by statistical significance (adjusted p‐value < 0.1) and categorized by log2 fold‐change direction. Genes with low expression (FPKM value < 50 as the cutoff threshold) were filtered out. Identified genes were mapped to the online database Database Annotation for Visualization and Integrated Discovery (DAVID).^[^ ^31^ ^]^ Kyoto Encyclopedia of Genes and Genomes (KEGG) pathway and Gene ontology (GO) analyses were performed to implement the enrichment analyses. Online platform SRplot^[^ ^62^ ^]^ was used for data visualization.

### Statistical Analysis

Data were analyzed using GraphPad Prism 10 (GraphPad Software, Inc., San Diego, CA, USA). Statistical significance was evaluated by one‐way or two‐way ANOVA, followed by post‐hoc tests. Detailed statistical information was provided in each legend, with p‐values indicated in the figures. Statistical significance was considered at P < 0.05. Experiments were performed at least three times. Detailed technical, biological replications and/or measurements were specified in each figure legend. Results were displayed as mean±SD.

## Conflict of Interest

The authors declare no conflict of interest.

## Author Contributions

The study was conceived and designed by YL.Z., Y.L., Y.H., HJ.G., and CX.Z.; in vitro experiments were conducted by YL.Z. and DW.L.; particle synthesis and characterization were performed by YL.Z., Y.H., and ZC.G.; bioinformatics analysis was carried out by YL.Z.; data analysis and interpretation were conducted by YL.Z., Y.H., and CX.Z.; the article was drafted by YL.Z. and revised by YL.Z., Y.H., Y.L., ZC.G., DW.L., HJ.G., and CX.Z. All authors read and approved the final version of the manuscript.

## Supporting information



Supporting Information

## Data Availability

The data that support the findings of this study are available from the corresponding author upon reasonable request.
