# Supplementary material for: Impact of Nanoparticle Stiffness on Endosomal Escape and Signaling Pathways in Cytosolic Delivery
Source: Adv Healthc Mater. 2025 Jul 14;14(28):2501706. doi: 10.1002/adhm.202501706 (PMC12581877; doi:10.1002/adhm.202501706)
Supplement: Supplementary file 1 — Supporting Information [file ADHM-14-0-s001.pdf]

ADVANCED HEALTHCARE MATERIALS

Supporting Information

for *Adv. Healthcare Mater.*, DOI 10.1002/adhm.202501706

Impact of Nanoparticle Stiffness on Endosomal Escape and Signaling Pathways in Cytosolic Delivery

*Yali Zhang, Yue Hui, Zichao Guo, Dawei Liu, Yun Liu, Huajian Gao and Chun-Xia Zhao**

Supplementary Materials for

Impact of nanoparticle stiffness on endosomal escape and signaling pathways in cytosolic delivery

Yali Zhang, Yue Hui, Zichao Guo, Dawei Liu, Yun Liu, Huajian Gao, Chun-Xia Zhao*

*Corresponding author. Email: chunxia.zhao@adelaide.edu.au

This PDF file includes:

Figures S1 to S17
Tables S1 to S3

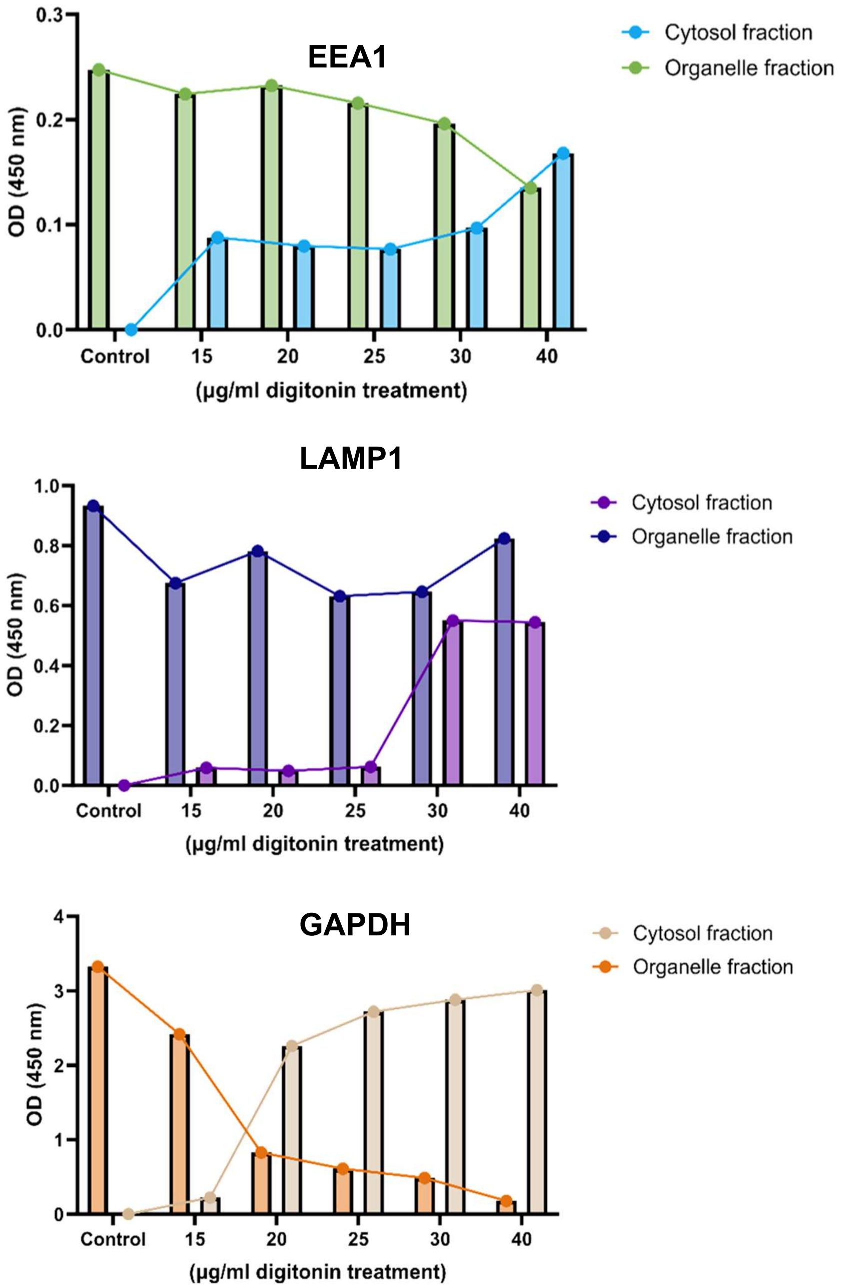

Fig. S1. ELISA analysis of cytosolic and organelle markers with various concentrations of digitonin. These analyses confirmed the separation of the cytosolic proteins glyceraldehyde 3-phosphate dehydrogenase (GAPDH) from the endosomal marker, early endosomal antigen 1 (EEA1) and lysosomal marker, lysosomal-associated membrane protein 1 (LAMP1) with an optimized concentration of digitonin as 25 μg/ml. (n=2)

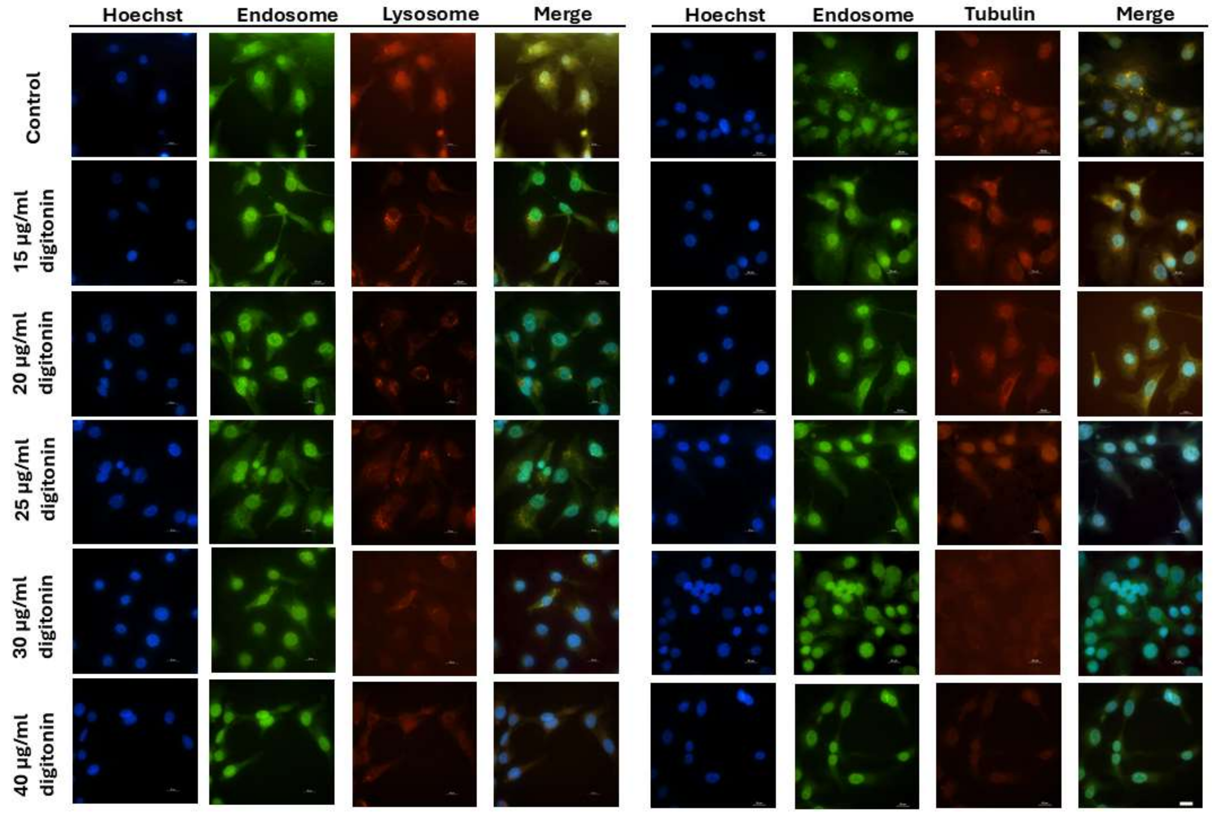

Fig. S2. Localization of cell compartments after digitonin treatment. Cells were stained before digitonin treatment using CellProbe endosome (green), LysoTracker (red) or tubulin (red). The nucleus is stained with Hoechst (blue). Scale bars, 20 µm.

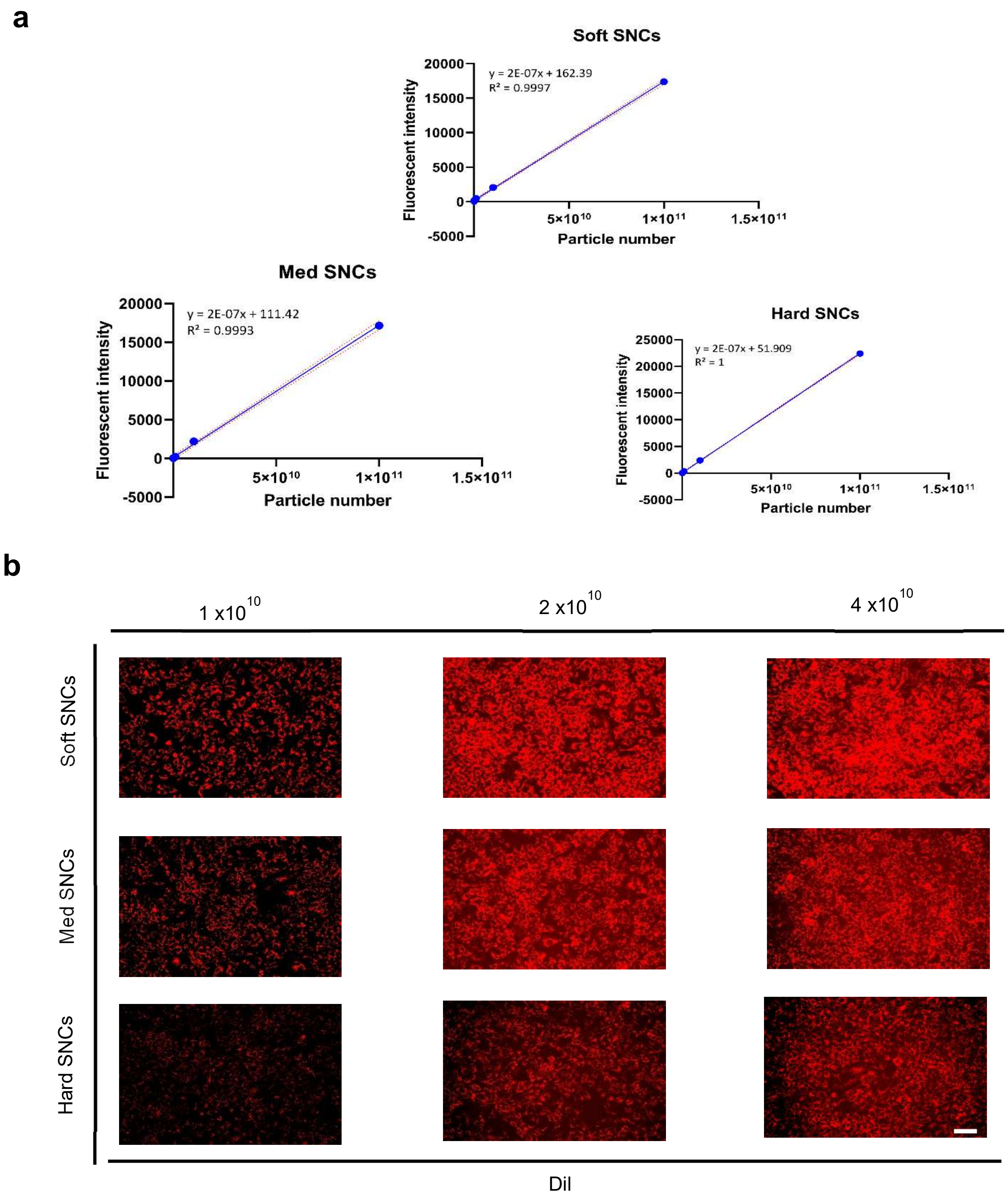

Fig. S3. The linear correlation between fluorescent intensity and particle concentration of SNCs. (a) Intracellular fluorescent intensity was acquired using a plate reader, and then corrected and normalized to non-treated controls. A linear relationship between particle concentration and fluorescent intensity was identified within a range from 1×10^6 to 1×10^{11} NPs (1×10^6, 1×10^7,

1×10^8, 1×10^9, 1×10^{10}, 1×10^{11}). Cell seeding density was 2×10^4 in 96-well plates. **(b)** Fluorescence visualization of Dil-loaded SNCs with various concentrations. Scale bars, 100 μm.

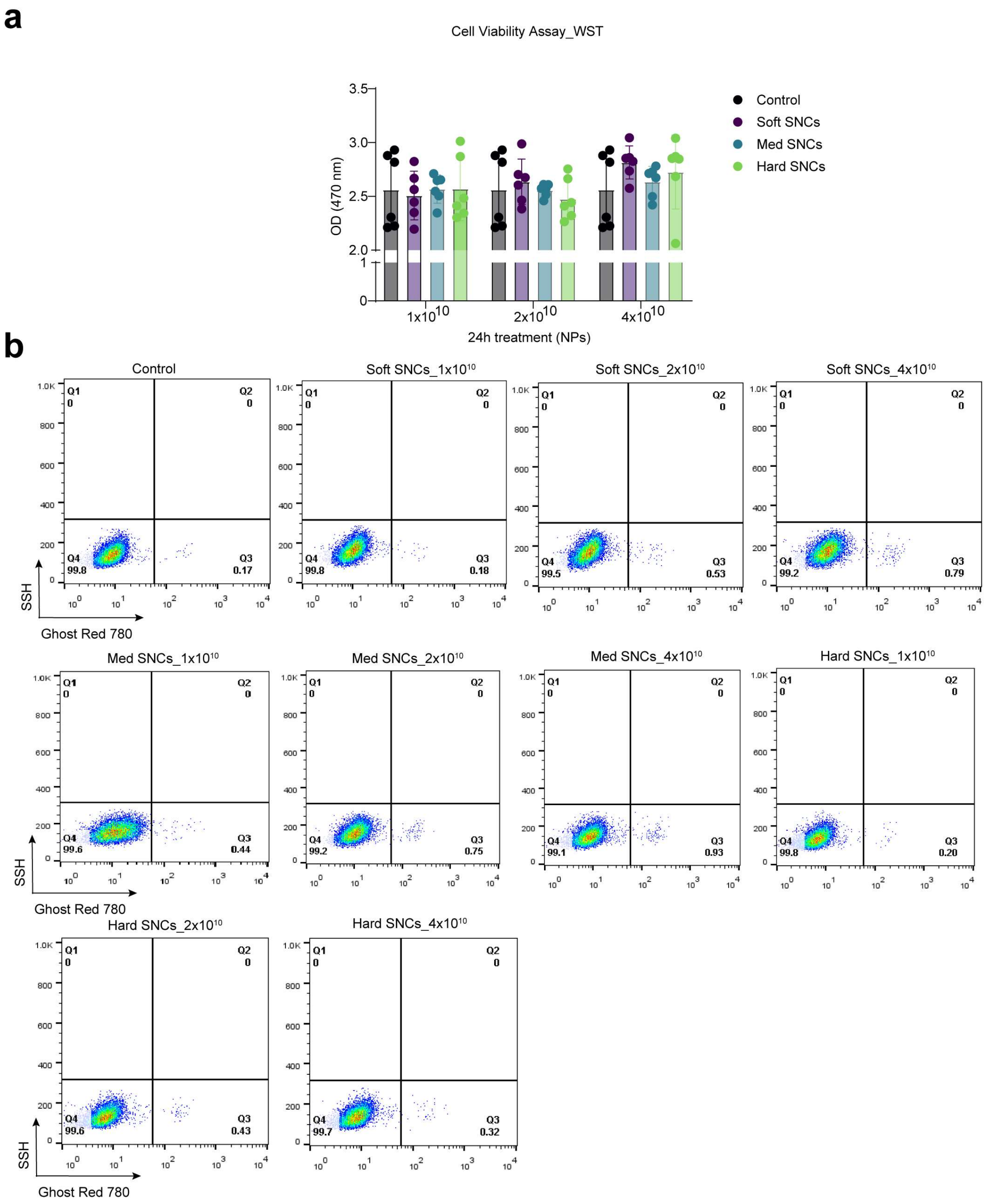

Fig. S4. Cell viability analysis of SNC samples with varying concentrations. (a) Cell viability WST assay with treatment of SNC samples. **(b)** Flow cytometry analysis of Ghost Red 780 assay with treatment of SNC samples. No significant changes of cell viability were observed. Data

represent as mean±SD. Statistical significance and P-values are determined using two-way ANOVA followed by Dunnett's multiple comparison test.

a

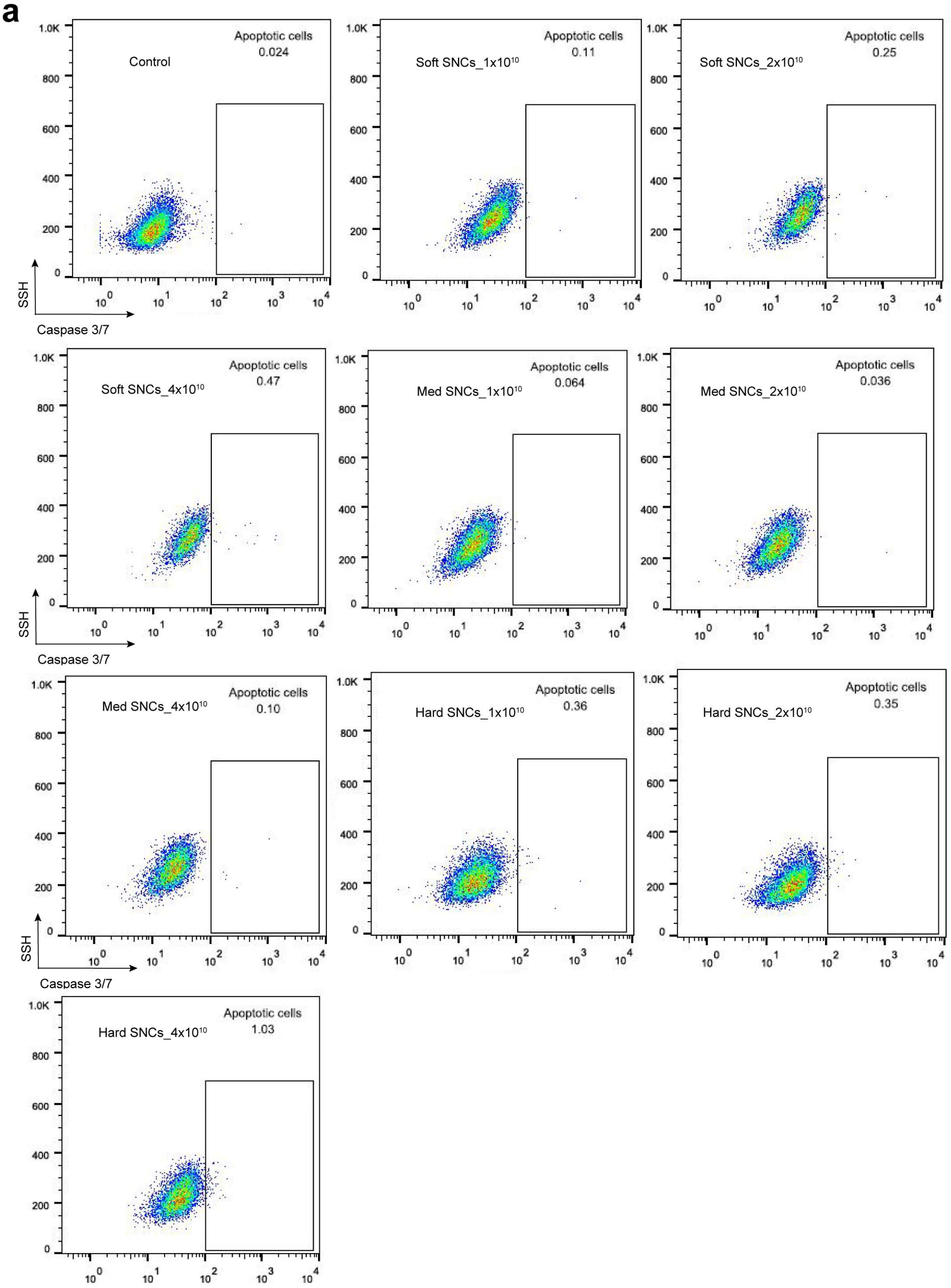

Fig. S5. Cell apoptosis analysis following SNCs treatment. (a) Flow cytometry analysis of apoptotic caspase 3/7 signals. **(b)** Representative images of caspase 3/7 with treatment of SNC

samples (scale bars, 500 μm). Cells were harvested for analysis after incubation with SNCs for 24 h.

Endosomal Escape Assay

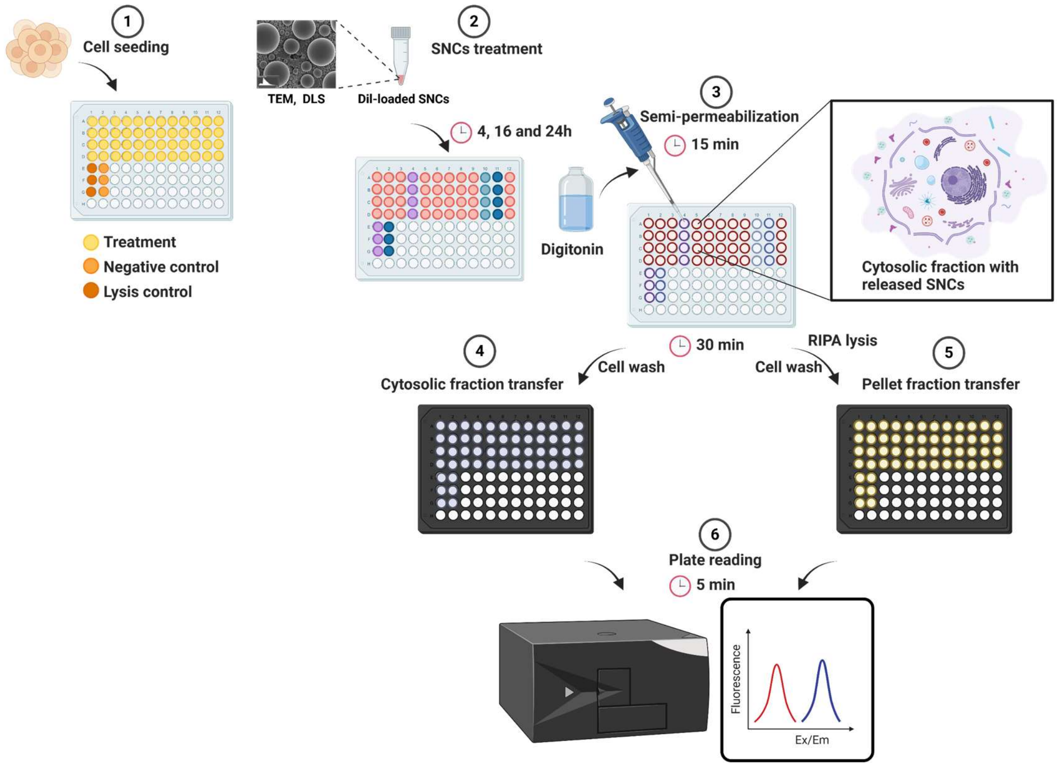

Fig. S6. Schematic illustration of the application of endosomal escape assay (EEA). (1) Cell seeding with 80% confluence. **(2)** Cell treatment with characterized and Dil-loaded SNCs for 4h, 16h and 24h. **(3)** Seimi-permeabilization with digitonin incubation. **(4)** Cell wash and cytosolic fraction transferred to fluorescent 96-well plate. **(5)** Cell lysis with RIPA incubation. **(6)** Fluorescence reading (Dil λex = 520 nm and λem = 565 nm) measurement. Part of the figure was created with BioRender.com.

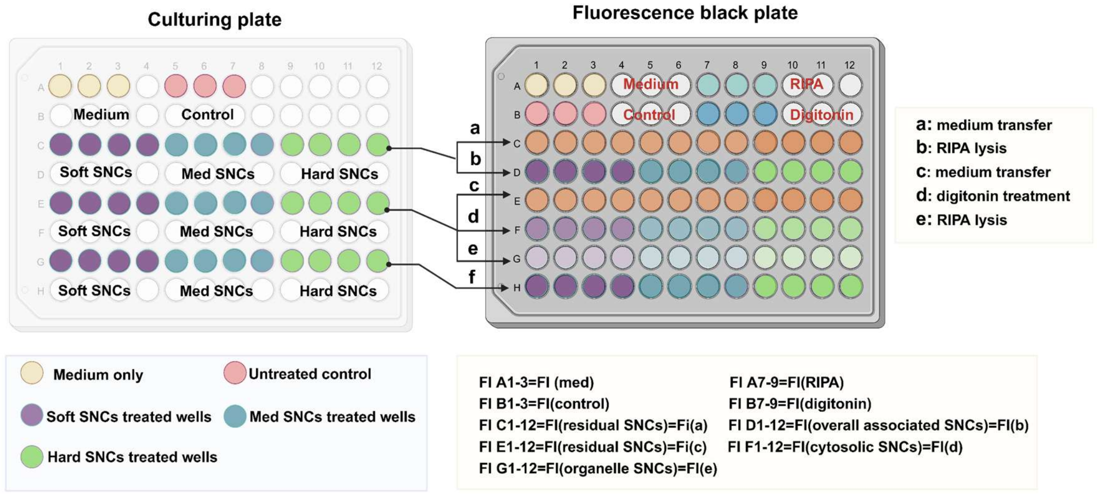

Fig. S7. Endosomal escape assay (EEA). Cells (2×10^4) cultured were seeded into 96-well culturing plates until 80% and then incubated with 2×10^{10} SNCs for 4, 16 and 24h, followed by treatments (a-e). All samples were then transferred to corresponding fluorescence black plates for fluorescence reading (Dil λex = 520 nm and λem = 565 nm). FI: Fluorescence intensity.

Equations for EEA:

FI (normalized overall SNCs) = FI (A):

$$[\boldsymbol{FI(a) - FI(med) + FI(b) - FI(RIPA)}]$$

FI (normalized uptake SNCs) = FI (B):

$$[\boldsymbol{FI(d) - FI(digitonin)}] + [\boldsymbol{FI(e) - FI(RIPA)}]$$

Cellular uptake efficiency (%):

$$\frac{FI(B)}{FI(A)} * 100$$

Endosomal escape efficiency (% of overall SNCs) $= \frac{\boldsymbol{FI(d) - FI(digitonin)}}{\boldsymbol{FI(A)}} * 100$

Endosomal escape efficiency (% of uptake) $= \frac{\boldsymbol{FI(d) - FI(digitonin)}}{\boldsymbol{FI(B)}} * 100$

a

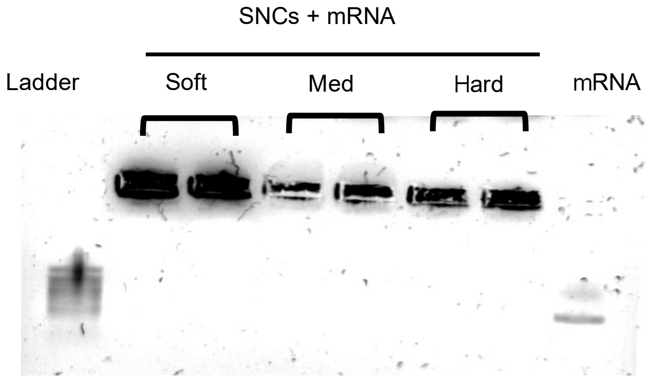

b

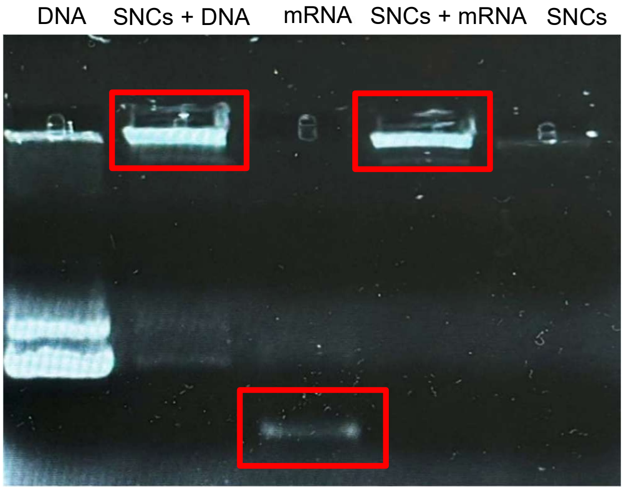

Fig. S8. Gel electrophoresis analysis of SNCs-mRNA. (a) Gel image of SNCs that were electrostatically bond with mRNA after 15 min incubation. No movement of SNC-mRNA was observed. **(b)** Gel image of SNCs, mRNA/DNA and SNCs-DNA samples after 25 min incubation.

a

Sample	Relative quantity	Mean Cq
1:10	1.61%	14.73
1:20	6.89%	12.63
1:30	23.28%	10.87
1:40	29.13%	10.55
control	100.00%	8.77

b

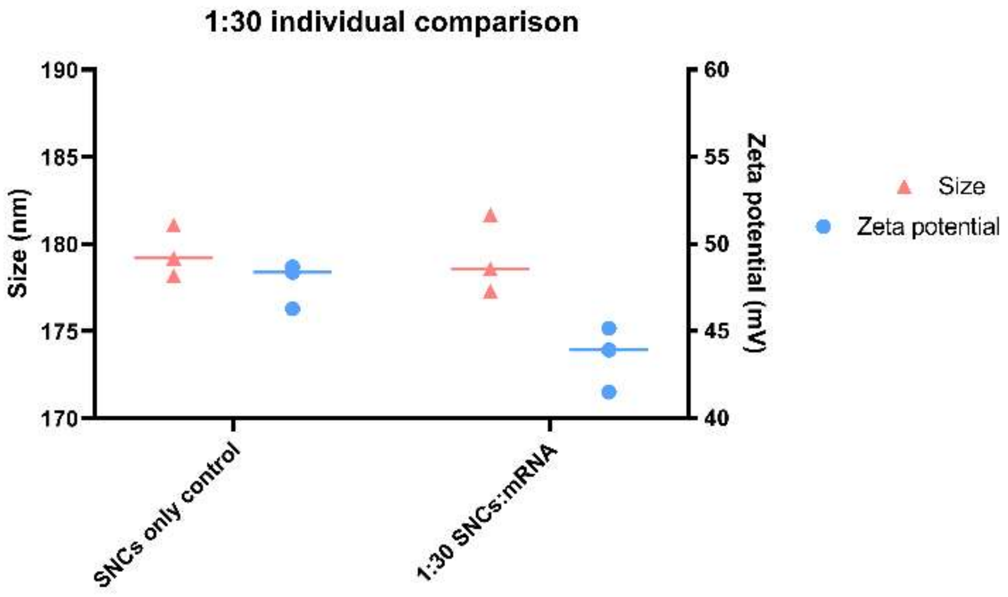

Fig. S9. Pre-tests to introduce mRNA-GFP to synthesized SNCs. (a) qPCR analysis was conducted following the SNCs-mRNA reaction. The SNCs number of treatments was 2×10^{10} NPs. **(b)** DLS analysis was performed after the SNC-mRNA reaction. SNCs across all stiffness levels were tested. A ratio of 1 SNC binding with 30 mRNA molecules was selected as the optimal ratio, as it led to a significant decrease in mRNA residues and zeta potential values of SNCs, and sufficient visualization of mRNA expression. SNCs-mRNA complex was suspended in HEPES buffer for characterization.

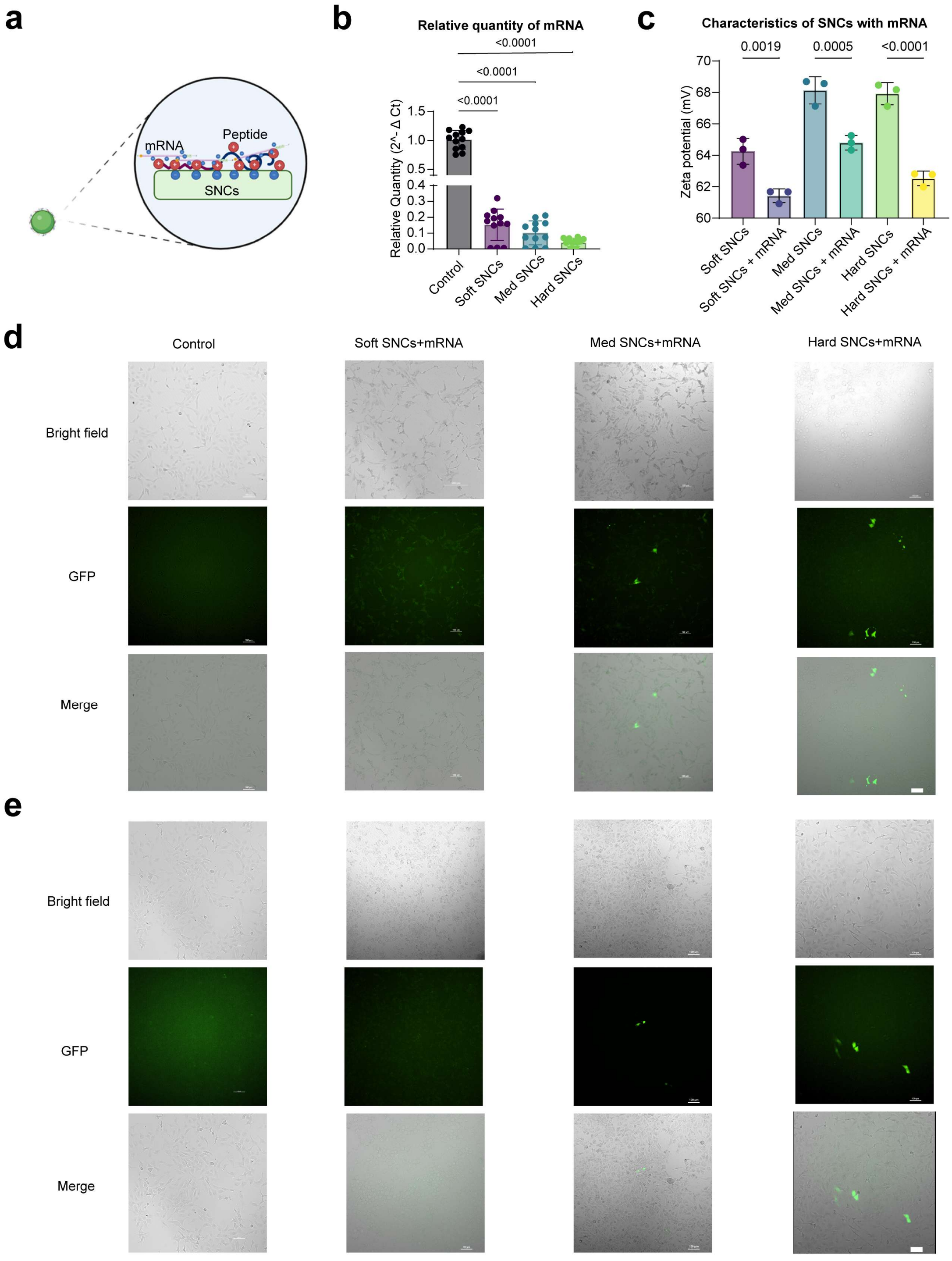

Fig. S10. Effects of stiffness on transfection efficiency. (a) Schematic attachment of mRNA with SNCs. **(b)** Relative quantity of residual mRNA following SNCs-mRNA incubation. mRNA only was applied as control. n=12. The complex ratio of SNCs and mRNA was applied as one NP binding with 30 mRNA molecules. **(c)** Comparison of zeta potential values of SNCs before and

after binding with mRNA. SNCs and mRNA were suspended in RNase-free water (to prevent mRNA degradation). **(d)** Representative fluorescence images of GFP expression following SNCs-mRNA treatment (scale bars, 100 μm). Cells treated with SNCs-mRNA for 4h and further incubation with fresh medium for 20h. **(e)** Representative fluorescence images of mRNA-GFP expression. Cells created with SNCs for 24h (scale bars, 100 μm). The hard SNCs showed greater transfection efficiency compared to softer counterparts. Data represent as mean±SD. Statistical significance and P-values are determined using one-way ANOVA followed by Tukey's multiple comparison test. Part of the figure was created with BioRender.com.

a

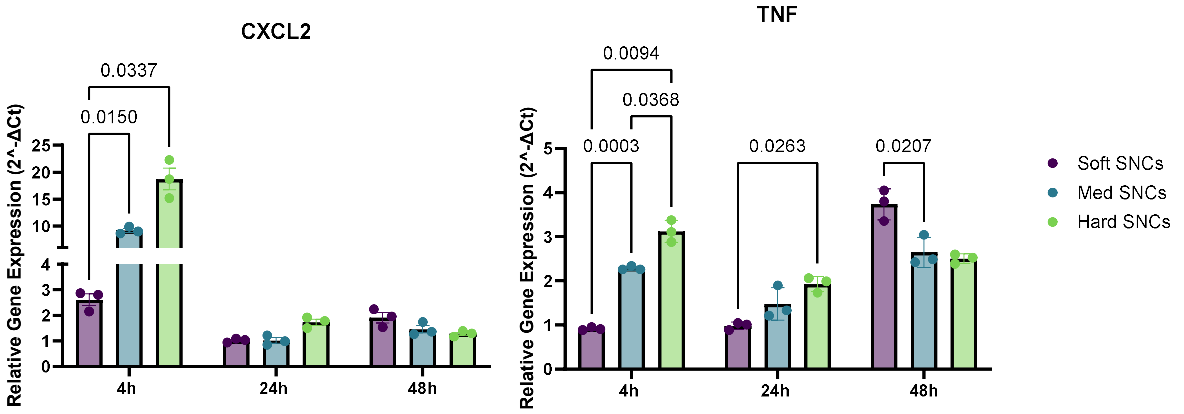

b

Repeat	A260/280	A260/230	RIN	Concentration (µg/µl)
		**Control**		
**Cont 1**	2.089	2.164	10	93.4
**Cont 2**	2.098	2.310	8.9	63.2
**Cont 3**	2.101	1.717	10	108.08
		**Soft SNCs**		
**S1**	2.042	1.754	8.4	60.68
**S2**	2.052	2.080	8.0	56.48
**S3**	2.072	2.173	10	103.76
		**Med SNCs**		
**M1**	2.069	2.233	10	96.48
**M2**	2.048	2.000	8.4	58.16
**M3**	2.086	2.099	10	91.60
		**Hard SNCs**		
**H1**	2.107	1.934	8.8	72.4
**H2**	2.085	1.966	9.0	78.32
**H3**	2.079	2.023	8.6	65.96

Fig. S11. Expression of stress markers after SNCs treatment. (a) Relative expression of CXCL2 and TNF after SNCs treatment. Cells were harvested for analysis at 4, 24 and 48h. **(b)** Summary of RNA quality and quantity submitted for sequencing. Data represent as mean ± SD. Statistical significance and P-values are determined using two-way ANOVA followed by Tukey's multiple comparison test.

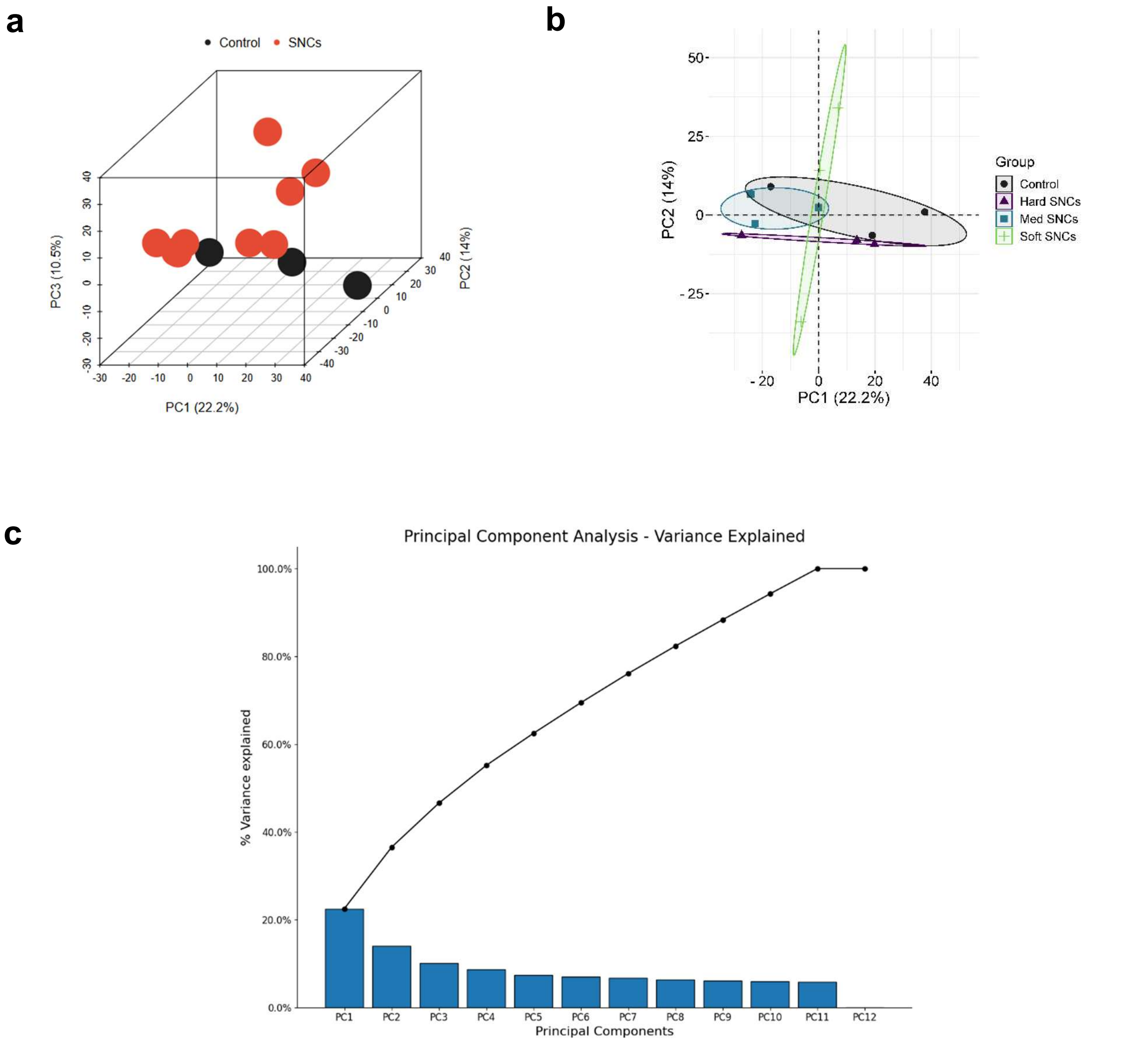

Fig. S12. PCA analysis of SNCs-induced transcriptomic changes. (a) PCA plot of SNCs regardless of stiffness and non-treated control. **(b)** PCA plot of SNC formulations and non-treated control. (c) Plot of total variance explained. ~50% of the total variance in the treatment response.

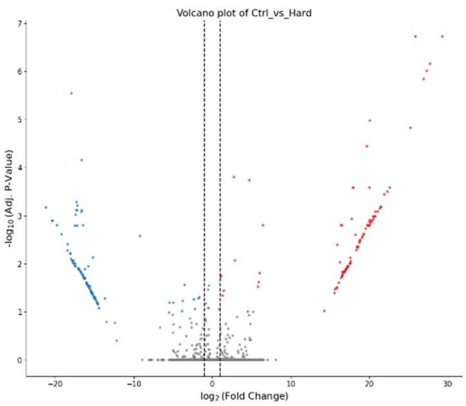

Control vs Hard SNCs

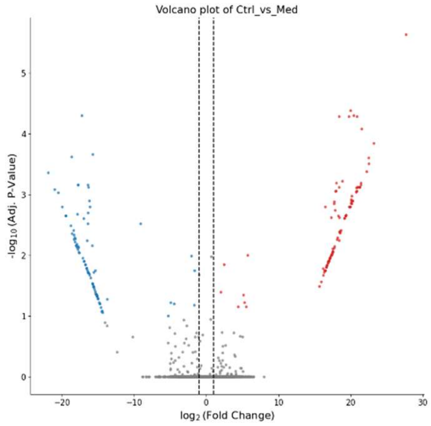

Control vs Med SNCs

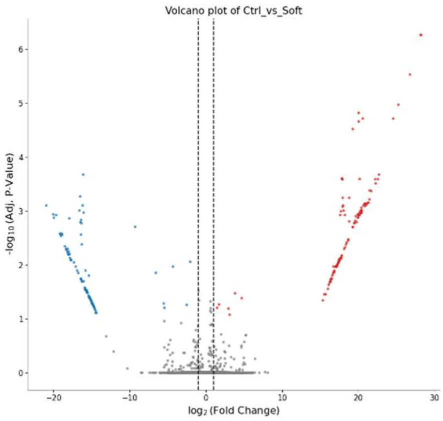

Control vs Soft SNCs

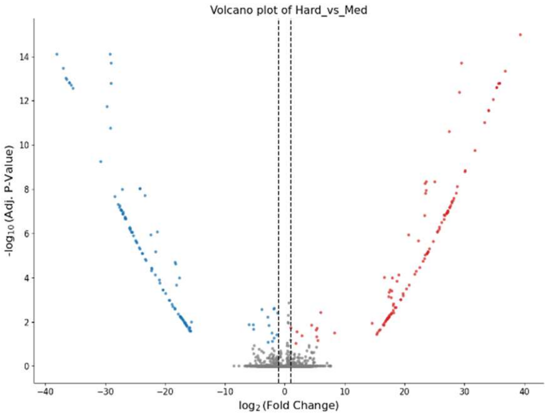

Hard vs Med SNCs

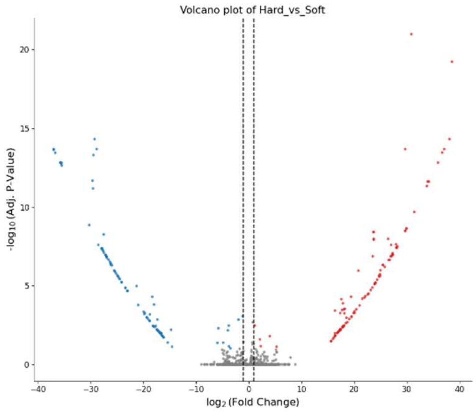

Hard vs Soft SNCs

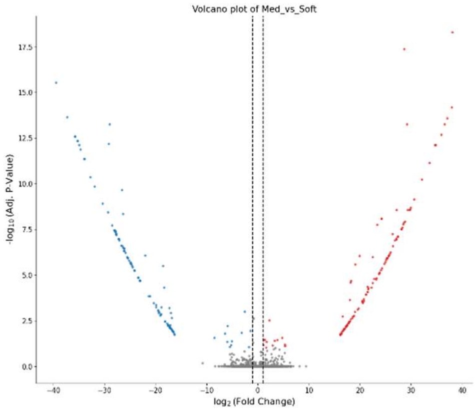

Med vs Soft SNCs

Fig. S13. Volcano plot of the differentially expressed genes (DEGs) in all compared settings. The DEGs were run with DESeq2 (data filtered by statistical significance adjusted p-value < 0.1 and split by log 2-fold-change direction).

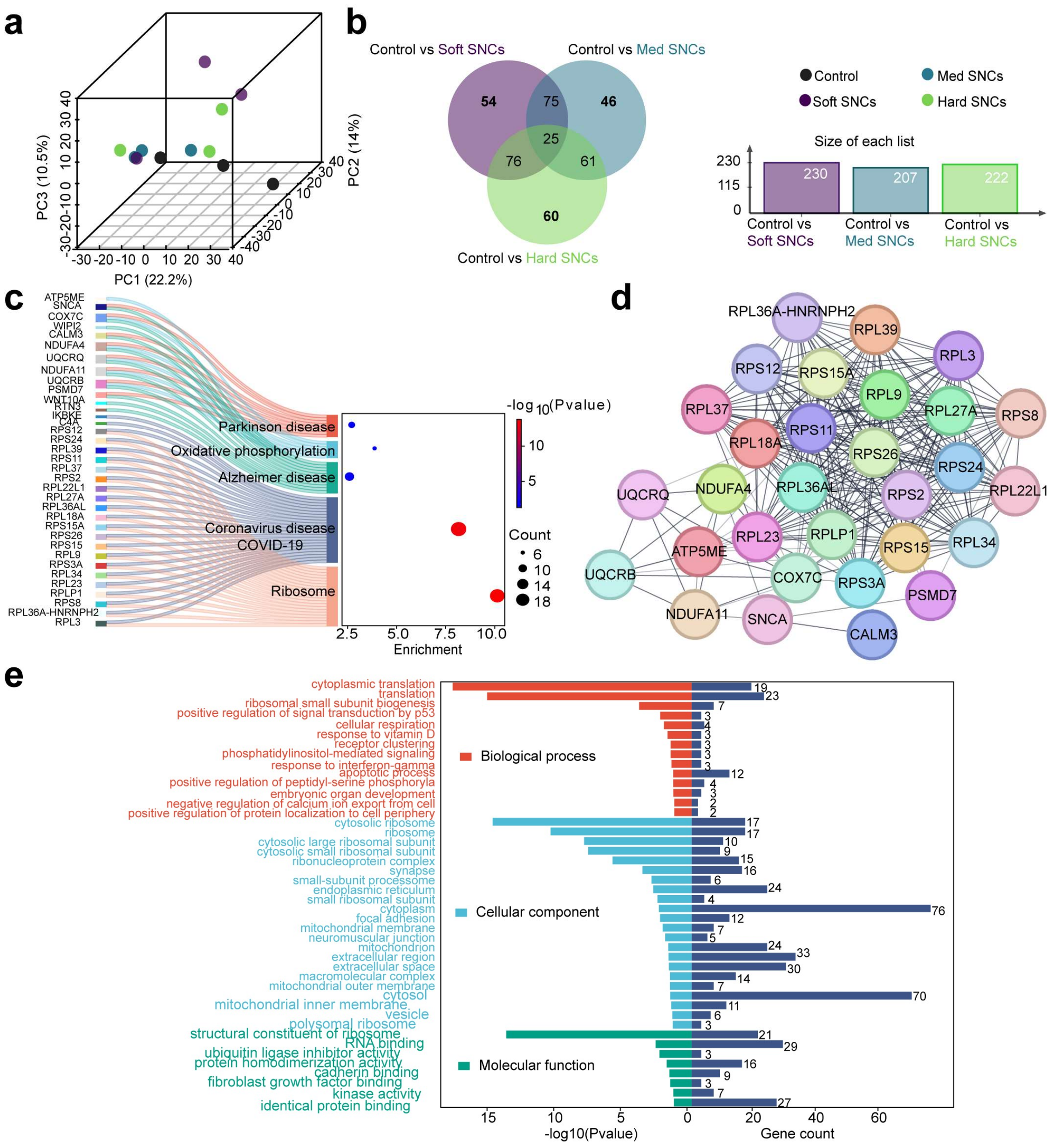

Fig. S14. Assessing SNCs-cell interactions. (a) PCA plot of SNCs-cell indicating the distinct clustering of SNCs and non-treated control. The first three principal components accounted for ~50% of the total variance in the treatment response as the nanomaterials used across this study are identical. **(b)** Venn diagrams of identified genes differentially expressed with SNCs treatment. **(c)** Enriched KEGG pathways identified from DEGs following SNCs treatment. **(d)** Schematic visualization of the STRING network generated by inputting the genes in significantly enriched KEGG pathways. Nodes (30) indicate features, and the edges (258) indicate predicted functional associations. **(e)** Enriched GO analysis of DEGs following SNCs treatment.

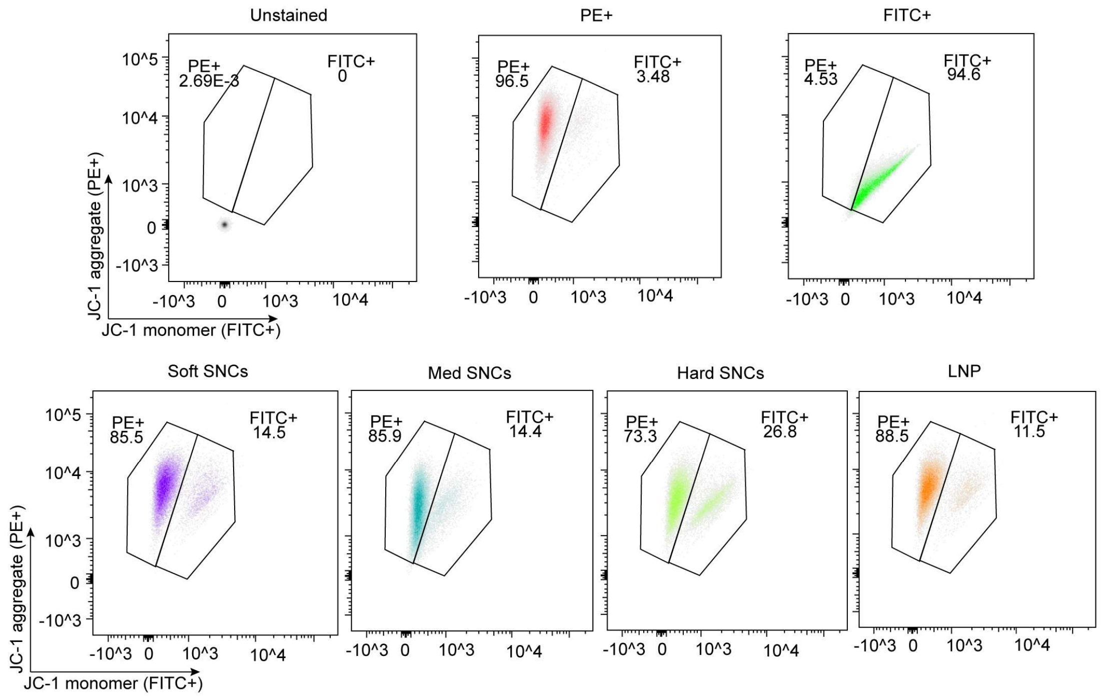

Fig. S15. Effect of NP stiffness on mitochondrial membrane potential (MMP). Flow cytometry dot plots JC-1 aggregate-area (PE+) vs JC-1 monomer area (FITC+). Cells (6×10^4) were treated with NPs (6×10^{10}) for 4h and further incubated for 16h with medium refresh. Cells were harvested for flow cytometry analysis at 24h. cells exposed to hard SNCs exhibited the highest green shift and lowest MMP among NP groups, with this effect being even more pronounced in the 24h assay.

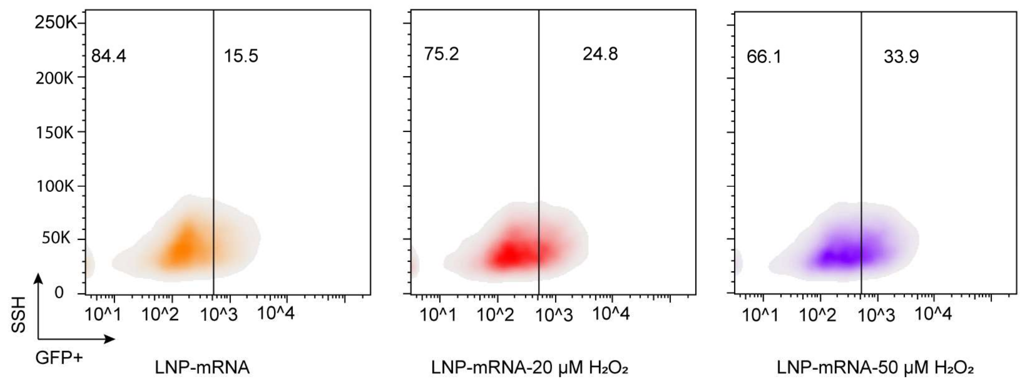

Fig. S16. Effect of ROS on NP transfection. Representative flow cytometry images of transfection efficiency (%) of LNP-mRNA with and without H_2O_2. Enhanced transfection efficiency (24.8% and 33.9%) was observed following H_2O_2 treatment (4h).

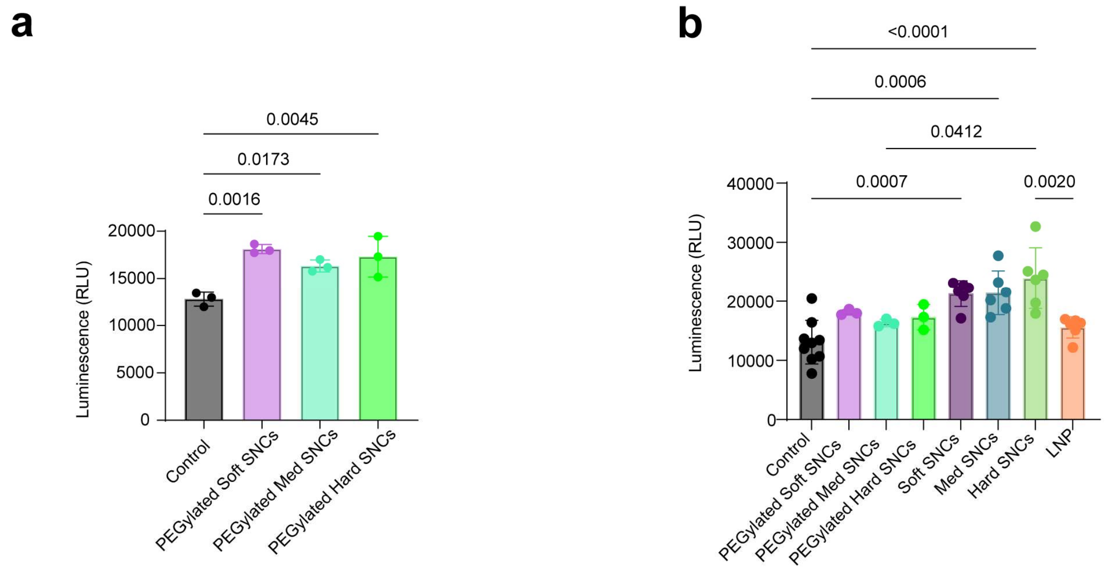

Fig. S17. ROS response after NPs treatment. (a) The level of ROS generation at 4h after treatment with PEGylated SNCs. Significant ROS levels were produced in all three PEGylated SNCs groups. **(b)** Combined comparison of SNCs with or without PEGylation. The naked SNCs induced greater ROS levels compared to control and LNPs. No statistically significant differences were identified in PEGylated SNCs compared to control and LNP as determined using one-way ANOVA followed by Tukey's multiple comparison test. Data represent as mean±SD.

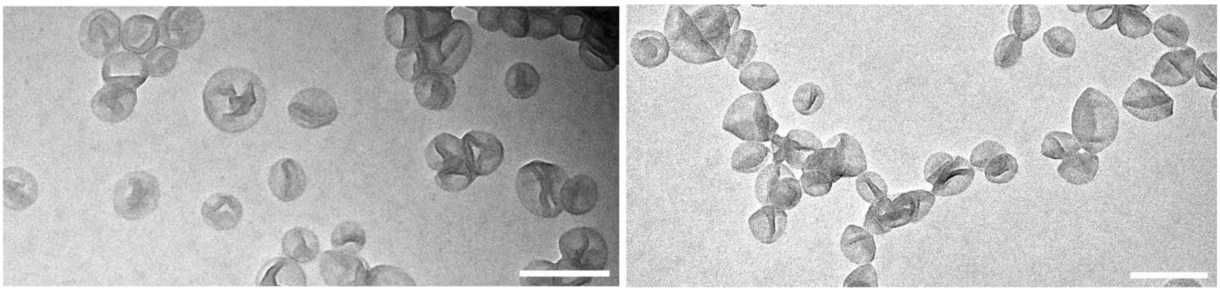

Fig. S18. TEM analysis of soft SNCs with/without mRNA. (left) Representative TEM images of the soft SNCs without mRNA (scale bars, 200 nm). (right) Representative TEM images of the soft SNCs with mRNA (scale bars, 200 nm).

Table S1. Primer sequences used for PCR

Gene	Forward Primer	Reverse Primer
**mRNA-eGFP**	ACTACCTGAGCACCCAGTCC	CTTGTACAGCTCGTCCATGC
**CXCL2**	CCAGTGCTTGCAGACCCT	TTCTTAACCATGGGCGATGC
**TNF**	GTCAACCTCCTCTCTGCCAT	CCAAAGTAGACCTGCCCAGA

Table S2. Dynamic light scattering measurements of synthesized SNCs and LNP

Characteristics	Soft SNCs	Med SNCs	Hard SNCs	LNP	LNP-mRNA
**Size (nm)**	169.77±1.452	175.60±0.816	180.00±0.374	80.83±1.886	90.52±0.962
**PDI**	0.1111±0.006	0.0621±0.006	0.0998±0.006	0.1529±0.002	0.1863±0.006
**Zeta potential (mv)**	44.20±0.445	41.58±0.366	39.27±0.449	-0.93±1.040	-8.395±0.473
**Young's modulus (MPa)**	1.37±0.22	13.22±0.85	1717±191	N/A	N/A

*10 µl of SNCs were suspended in 990 µl of 2.5 mM HEPES buffer, pH 7.5

*20 µl of LNP were suspended in 980 µl of 1× PBS buffer for size and PDI characterization; 50 µl of LNP were suspended in 950 µl of MilliQ-water for zeta potential characterization

Table S3. **Dynamic light scattering characterization of PEGylated SNCs**

	PEGylated Soft SNCs	PEGylated Med SNCs	PEGylated Hard SNCs
**Size (nm)**	238.8±7.21	266.1±5.79	353.6±9.49
**PDI**	0.1697±0.013	0.1242±0.013	0.1788 ± 0.010
**Zeta potential (mv)**	-1.254±0.167	-1.038±0.052	-0.665±0.091

*10 µl of SNCs were suspended in 990 µl of 1× PBS buffer
